# Affibody-based optical imaging probe for noninvasive detection of liver fibrosis

**DOI:** 10.7150/thno.117262

**Published:** 2026-01-01

**Authors:** Raana Kashfi Sadabad, Paul Stephen Marinec, Lawrence Lechuga, Robert Thompson, Ran Cheng, Ashmita Saigal, Irodiel Vinales, Salomon Martinez, Allan Eduardo Solis, Emily Melody Tso Newman, Una Goncin, Christopher Novotny, Corey Miller, Chih-Liang Chin

**Affiliations:** 1 Merck & Co., Inc., Rahway, NJ, USA.; 2 Department of Chemistry, Boston University, Boston, MA 02215, USA.; 3 Department of Chemistry and Biochemistry, University of Texas at El Paso, El Paso, Texas 79968, USA.

**Keywords:** miniprotein, Affibody molecules, imaging probe, MASH, fibrosis, fluorescence imaging, optical imaging

## Abstract

**Background:** Metabolic dysfunction-associated steatohepatitis (MASH) is a progressive liver disease affecting 2-6% of the global population, which can eventually lead to liver failure or cancer. The current standard for diagnosis is liver biopsy, which is invasive, subjective, painful, and carries potential complications. We present a novel non-invasive optical imaging approach using a miniprotein based probes (affibody) targeting collagen type-1 (COL-1), a histologically established marker of MASH, to detect liver fibrosis in animal models of MASH *in vivo* with high sensitivity and specificity. thereby enabling the potential of early diagnosis and longitudinal assessment of disease progression and response to treatments.

**Methods:** We synthesized multiple affibody molecules with varying binding affinities to COL-1, two of which (called Clone 3 and 7) are reported here, and a non-binding control structure. We conjugated the affibodies with a near-infrared (NIR) fluorophore dye (Dy800), enabling the detection and monitoring of the probes *in vivo* using fluorescent optical imaging. We used two different MASH animal models, high-fat choline-deficient (HFCDA) diet-fed mice and Gubra-Amylin (GAN) mice, along with their age-matched normal chow-fed (NC-fed) controls, to assess the biodistribution, binding specificity, and liver accumulation of the COL-1 affibodies through *in vivo* whole-body optical imaging and *ex vivo* tissue imaging.

**Results:** The affibody optical imaging probe had 50-fold higher affinity (~34 nM) compared to the state-of-the-art imaging probes (typically, > 1 µM). It demonstrated rapid blood clearance via renal elimination, with complete elimination observed at 48 h post-injection. Imaging data obtained from both animal models showed significantly higher liver fluorescence signal intensity compared to their age-matched NC-fed controls. Specificity was confirmed by comparison of images collected with binding and non-binding probes, the latter showing significantly lower accumulation in the liver. A strong correlation between histology-derived collagen content and *ex vivo* liver imaging data was found in diseased animals (r^2^ = 0.86).

**Conclusions:** *In vivo* imaging of liver fibrosis using collagen-targeting affibody probes offers a promising non-invasive alternative to liver biopsy, potentially improving diagnosis accuracy and accelerating drug development.

## Introduction

Fibrosis is a common pathological process that affects nearly all major organs in the human body, leading to underlying chronic conditions, such as hepatic, pulmonary, renal, and cardiovascular diseases 1. It is estimated that fibrotic diseases account for up to 45% of mortality in developed Western countries 2, and 16.5% of all global deaths in 1990. This proportion steadily increased to 17.8% by 2019 3. There are several promising anti-fibrotic drugs; however, their therapeutic benefit has been limited to slowing, but not reversing the progression of fibrosis 4. Metabolic dysfunction-associated steatohepatitis (MASH) is a chronic, progressive liver disease caused by excess fat accumulation in the liver (steatosis). Around 60% of steatosis cases lead to chronic inflammation, 41% of which may further develop liver scarring or fibrosis, 22% of which can advance to liver cirrhosis 5, 6. Early diagnosis is crucial for effectively managing diseases and preventing disease progression. Without timely diagnosis and treatment, the disease can progress to liver failure and cancer 7, 8. As of today, only one drug (resmetirom) has been approved by the U.S. Food and Drug Administration (FDA) for the treatment of MASH, while other experimental therapeutics have remained largely ineffective 9. Current diagnosis of fibrosis relies on liver biopsy, which is invasive, prone to sampling bias, painful and not well-tolerated for repetitive testing, and carries risks of complications 10, 11. As a non-invasive alternative, *in vivo* imaging techniques such as CT, ultrasound, and MRI have been used to detect fibrosis in the lungs, heart, and liver 4. While identifying advanced or late-stage pathological features of liver fibrosis, these imaging techniques are not sensitive enough for early-stage diagnosis and moreover, they often do not provide any insight into the molecular underpinnings of the diseases 12-14. An alternative approach to address these limitations is to use molecular imaging probes that specifically target tissue fibrosis markers.

The excessive deposition of extracellular matrix (ECM) components, such as elastin and collagen, is directly related to the progression of fibrosis 12. COL-1 is a key component of the ECM, with its concentration increasing significantly—often more than tenfold—in fibrotic conditions 15. Additionally, the extracellular localization of COL-1 facilitates its accessibility to imaging probes, which makes COL-1 highly suitable for detection using imaging systems combined with targeted probes. COL-1 concentration changes dynamically during disease progression, regression, and in response to antifibrotic drugs 16. Imaging probes such as peptides, or nanoparticles that selectively bind to COL-1 have been developed 12, 17. For example, EP-3533 and its modified variant CM-101 are cyclic peptides 18, 19, with moderate affinities for COL-1 ranging from 1.8 to 7.4 µM, that have been explored for imaging idiopathic pulmonary fibrosis (IPF) 20, cardiac fibrosis 21, and liver fibrosis 20, 22. However, EP-3533 utilized gadopentetate dimeglumine (Gd-DTPA) as a chelate, leading to gadolinium retention in tissues and an associated risk of nephrogenic systemic fibrosis 23. To overcome these limitations, EP-3533 was radiolabeled with ^68^Ga using a DOTA chelator to produce ^68^Ga-CBP8 24. This modification allowed for a lower dose with reduced toxicity and yielded promising results in preclinical models of lung fibrosis 25. Another example is ProCA32.collagen1, a protein-based MRI contrast agent that uses the same COL-1 targeting peptide in EP-3533 and CM-101. It offered an alternative solution to reduce the metal toxicity associated with gadolinium, exhibiting a slightly better affinity of 1.42 µM for COL-1 and demonstrating increased uptake in fibrotic livers in MASH animal models. However, due to its larger size, it resulted in longer blood circulation and delayed imaging after probe injection 26. While both ^68^Ga-CBP8 and ProCA32.collagen1 are promising, our goal is to demonstrate the feasibility of developing imaging probes with improved COL-1 affinities to enable effective binding at significantly lower doses to further minimize the risk of toxicity.

Small miniprotein scaffolds (58 amino acid peptides, 6-7 kDa), called affibodies, can be engineered to bind to a variety of target proteins with nanomolar affinity, and are gaining popularity in molecular imaging. Their small size enables better tissue penetration and quicker clearance from the body compared to larger scaffolds such as antibodies 27, 28. This scaffold also enables excretion through the renal system without retention in the liver, making such small probes suitable candidates for liver imaging 29, although we are not aware of affibodies being used for MASH prior to this study. In this study, we engineered multiple affibody variants based on the Z-structural domain of *Staphylococcus* protein A with nanomolar binding affinity and high specificity for human COL-1, and conjugated them with a NIR fluorophore dye (Dy800) to enable *in vivo* imaging in HFCDA and GAN mouse models of MASH. We screened seven different affibody candidates (clones 1 to 7) and selected two of them with the highest affinity (Clone 3 and 7) for subsequent assessment of their biodistribution, binding specificity, and liver accumulation using pre-clinical *in vivo* whole-body 2D and 3D optical imaging as well as *ex vivo* tissue imaging.

## Materials and Methods

### COL-1 binding affibody production-Phage Panning

Naïve mini-protein phage libraries based on the 58-residue three-helix Z domain were constructed as previously described 30. Phage selections were performed using biotinylated human COL1A1 protein (Acro) captured with Pierce streptavidin magnetic beads (ThermoFisher). Several rounds of panning were performed with decreasing amounts of antigen until a consensus set of binders was isolated.

### Phage binding ELISAs

To evaluate the relative binding affinity of the affibodies for COL-1, we performed ELISA assays. Briefly, dilutions of affibody molecules phage binders labeled with enzyme horseradish peroxidase (HRP) were added to plates coated with human COL-1. The HRP enzyme catalyzes a reaction with the chromogenic substrate (3,3′,5,5′-Tetramethylbenzidine (TMB) in this study), resulting in a color change. The color change was then measured using a spectrophotometer. The color intensity r is proportional to the enzyme activity, which directly correlates with the binding of the affibody to COL-1. 96-well Maxisorp plates (Nunc) were coated with 100 µL/well of NeutrAvidin (ThermoFisher) at 2.0 mg/mL in 1X PBS + 0.1% Tween [PBST] and incubated overnight at 4°C. The next day, the plates were washed three times with PBST and incubated with 100 µL/well biotinylated human COL1A1 (Acro) at 1.0 mg/mL for 30 minutes at room temperature with shaking at 800 rpm. Plates were washed three times with PBST and serial dilutions of the phage pools normalized to an OD269 = 1.0 were added to the plates and incubated for 15 minutes at room temperature. Phage were detected by incubating with a 1:5000 solution of anti-M13-HRP (ThermoFisher) for 30 minutes at room temperature with shaking at 800 rpm. After washing the plates three times with PBST, 100 µL of SureBlue TMB substrate (SeraCare) was added allowing the color to develop for 3-5 minutes. The reaction was quenched with KPL TMB stop solution (SeraCare) and the optical density (OD at 650 nm) was recorded using a plate reader. Graphs were generated using GraphPad Prism to rank order clones for chemical synthesis.

### Synthesis and analysis of COL-1 binding affibody molecules

The peptides were synthesized using Fmoc-based Solid Phase Peptide Synthesis (SPPS), which included resin loading, Fmoc deprotection, coupling with Fmoc-Lys(Boc)-OH, and acetylation, followed by TFA cleavage and precipitation. The crude peptides were purified by semi-preparative reverse-phase HPLC, and the final products were obtained by lyophilization. The peptides were analyzed by LC/MS to confirm their molecular weight and purity. Full experimental details are provided in the supporting information.

### Synthesis and purification of Dye800-Labeled affibody molecules

Maleimide chemistry was used for site-specific labeling of COL-1 binding and non-binding affibody molecules with IRDye800CW-Maleimide. Briefly, a 1:1 molar ratio of affibody molecule and IRDye800CW-Maleimide was dissolved in a 1:1 mixture of MeCN and water, with the pH adjusted to 8 using 0.5 M NaHCO₃. The mixture was stirred at 20 °C for 30 min, and LC-MS confirmed its completion. The pH was then adjusted to 6 using 1 M HCl. Semi-preparative reverse phase HPLC was performed on a YMC-Actus 10 μm C18 column (30 mm x 250 mm) (GLX-281). Separations were achieved using linear gradients of buffer B in A (Mobile phase A: water containing 0.075% TFA, mobile phase B: Acetonitrile (ACN)), at a flow rate of 20 mL/min (preparative). The gradient was 19-39-40 min, and the retention time was 31 min.

The site-specific conjugation of Clone 7 affibody (MW: 8167.05 Da) with IRDye800CW-Maleimide yielded 15.88 mg of purified green product (93.4% purity), corresponding to a reaction yield of 53%. Low-resolution LC/MS of purified Clone 7-Dye800 gave 9 charged states of the peptide: [M+5H]^5+^ = 1634.6; [M+6H]^6+^ = 1362.2; [M+7H]^7+^ = 1167.8; [M+8H]^8+^ = 1022.0; [M+9H]^9+^ = 908.5; [M+10H]^10+^ = 817.9; [M+11H]^11+^ = 743.8; [M+12H]^12+^ = 681.7; [M+13H]^13+^ = 629.2. The experimental mass agrees with the theoretical mass of 8167.05 Da [M+1].

For the non-binding-dye conjugate clone, the same reverse-phase HPLC conditions were used (identical column, mobile phases, and flow rate) with a gradient of 35-55-40 min, and the retention time was 32 min. The conjugation yielded 15.08 mg of product (96.9%), corresponding to a 45.3% reaction yield based on the initial 4.44 µmol of starting material. 8 charged states of the peptide: [M+4H]^4+^ = 1874.4; [M+5H]^5+^ = 1500.0; [M+6H]^6+^ = 1249.9; [M+7H]^7+^ = 1072.0; [M+8H]^8+^ = 938.1; [M+9H]^9+^ = 833.6; [M+11H]^11+^ = 862.7; [M+12H]^12+^ = 681.7; [M+13H]^13+^ = 625.4 was obtained by low-resolution LC/MS. The experimental mass agrees with the theoretical mass of 7494.23 Da [M+1].

### Thermal stability and particle size analysis

We used the Prometheus Panta system (NanoTemper Technologies) to evaluate thermal stability and aggregation behavior of the affibody. Samples were prepared in PBS buffer at a concentration of 1 mg/mL. Thermal unfolding and refolding transitions were monitored by intrinsic tryptophan fluorescence (emission ratio at 350/330 nm) and back reflection optics to detect changes in protein aggregation. The temperature ramp ranged from 15 °C to 95 °C at a constant heating rate of 1 °C/min. Melting temperatures (Tm) corresponding to protein unfolding and refolding were determined by the first derivative of the fluorescence ratio. Dynamic Light Scattering (DLS) measurements were simultaneously collected during the thermal ramp to determine the cumulative hydrodynamic radius (nm), showing the colloidal stability. All measurements were conducted in triplicate, and PR.ThermControl software was used to analyze the data.

### Animals

Procedures involving the care and use of animals in this study were reviewed and approved by the Institutional Animal Care and Use Committee at Merck & Co., Inc., Rahway, NJ, USA (protocol# 400180). During the study, the care and use of animals were conducted in accordance with the principles outlined in guidance from the Association for Assessment and Accreditation of Laboratory Animal Care, the Animal Welfare Act, the American Veterinary Medical Association Euthanasia Panel on Euthanasia, and the Institute for Laboratory Animal Research Guide to the Care and Use of Laboratory Animals.

We used two dietary models to induce MASH:

*Gubra Amylin NASH (GAN) Diet:*

In this study, 28 C57BL/6 male mice, aged 6-7 weeks were used. Mice were evenly divided into two groups: one received a Gubra-Amylin NASH diet (Research Diets, D09100310; 40% fat kcal, 22% fructose, and 2% cholesterol) and the other received an NC diet (Lab5001, Research Diets). The feeding regimen lasted for 31 weeks.

*High-Fat, Choline-Deficient, and L-amino Acid-defined (HF-CDA) Diet:*

Seven-week-old male C57BL/6J mice were obtained from Jackson Laboratory and acclimated for approximately one week. To establish liver fibrosis, mice were fed the HFCDA diet (Cat# A06071302, Research Diets) for 8 weeks, with imaging performed at week 8, while healthy mice were fed an NC diet. Before imaging, each diet group was divided into three subgroups, each receiving one of three probes: Clone 7 (Kd = 34 nM), Clone 3 (Kd = 554 nM), or the non-binding probe.

The time points for each model were selected based on the known kinetics of fibrosis development. In the HFCDA model, substantial fibrosis is observed as early as 8 weeks, as demonstrated in previous studies 31. In contrast, the GAN model typically requires a longer duration to develop advanced fibrosis features, with 31 weeks commonly used in C57BL/6 mice according to established protocols 32-34. These durations were chosen to ensure each model reached a relevant disease stage for imaging and evaluation.

**Scheme 1:**
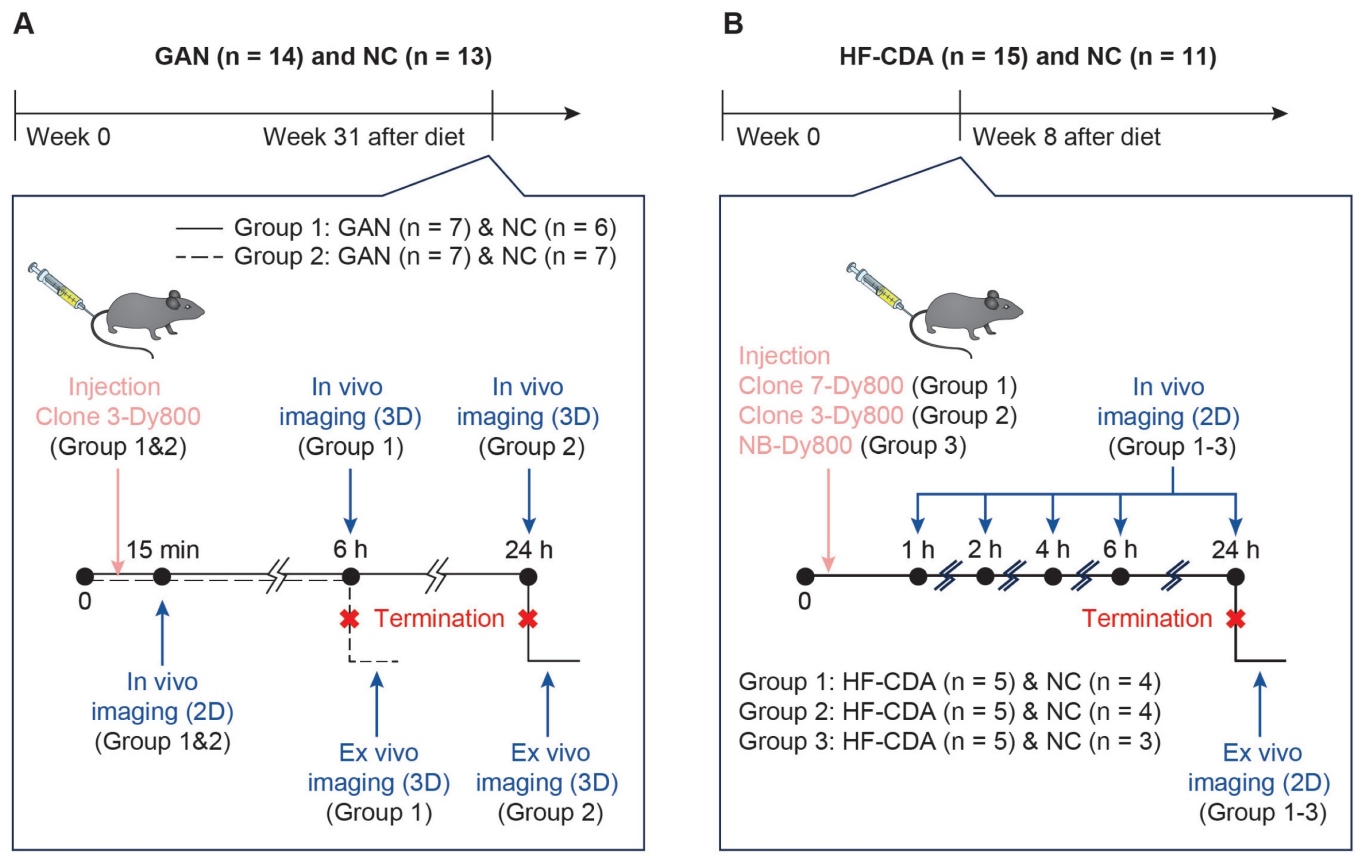
** Study design for *in vivo* imaging of liver fibrosis using affibody probes.** A) Mice were fed GAN or NC diets for 31 weeks before imaging. Mice were divided into two groups and injected with Clone 3-Dy800. *In vivo* 3D fluorescence imaging was performed at multiple time points (15 min, 6 h, and 24 h) followed by termination and *ex vivo* imaging. B) Mice on HFCDA diet or NC diet were imaged at week 8. Three probes (Clone 7-Dy800, Clone 3-Dy800, NB-Dy800) were injected in separate groups. *In vivo* imaging was performed at 1-24 h post-injection, followed by termination and *ex vivo* 2D imaging.

Mice in each subgroup were dosed with 1.5 mg/kg of the respective probe. Experimental design, animal number, and group assignments are shown in Scheme 1.

### *In vivo* optical imaging

The *in vivo* biodistribution and liver accumulation of COL-1 affibodies in GAN and HF-CDAA mice were assessed using micro-computed tomography-fluorescence tomography (μCT-FLT) (MILabs B.V., Utrecht, the Netherlands) and 2D epi-illumination fluorescence imaging (FLI) (IVIS SpectrumCT (Revvity). Prior to acquisition of *in vivo* fluorescence images, mice were transitioned to a low-fluorescence AIN-76 diet (D09100310 non-irradiated diet, Research Diets, Inc) four days prior to imaging to minimize auto fluorescence signal. The abdominal region was shaved to reduce optical signal attenuation near the liver. For 3D imaging, both the back and abdominal areas were shaved to minimize signal interference from hair. The probe was then administered via tail vein injection and the animals were anesthetized with isoflurane at a concentration of 3-3.5%. For μCT-FLT images, mice were placed in the prone position between two acrylic glass plates in the animal holder, where a heating element and 1.5-2% isoflurane were used to maintain both temperature and anesthesia throughout the scan. The entire setup was positioned between the FLT laser and a cooled CCD camera. Photographic images were acquired to help locate anatomic landmarks for field of view (FOV) selection. The FOV was selected to include the base of the mouse tail to the neck. Between 100-110 scan points were acquired using a laser and a filter with excitation and emission wavelengths at 730 nm and 794 nm, respectively. After completing the optical portion of the FLT scan the total body μCT scan was automatically started, without the need to reposition the animal. After imaging, mice were promptly removed and allowed to recover. Prior to injection of either COL-1 binding or non-binding probes, baseline imaging was performed in a subset of the NC-fed and GAN-fed mice to assess auto fluorescence in the organs of interest. Imaging was also performed immediately after injection (t = 15 min), as well as at 6- and 24-hs post-injection, to longitudinally assess the biodistribution of the fluorescent probe. Given the large number of mice, the imaging sessions were conducted over two identical sessions, separated by one week. The animals were sacrificed at 6 and 24 hs and their organs were excised for *ex vivo* evaluation via IVIS system in 2D reflectance imaging mode with a 745 nm and 800 nm excitation and emission wavelengths, respectively.

For 2D FLI, mice were placed in the supine position with the largest field of view selected. The exposure time and sensitivity settings were automatically selected to optimize signal detection. Mice were imaged before probe injection to establish a baseline, and at multiple time points (1, 2, 4, and 24 hs) following probe injection. The imaging was performed using an excitation filter at 745 nm and an emission filter at 800 nm. Fluorescence signal intensity was quantified using average radiant efficiency [p/s/cm²/sr] / [µW/cm²] within regions of interest (ROIs) placed over the chest area. The background signal from pre-injection images was subtracted for normalization. All 2D FLI images were analyzed using Living Image software.

### FLT image analysis and statistics

All CT datasets were reconstructed at 10 µm voxels. Resultant CT volumes were then used to reconstruct the 3D FLT dataset using the GPU-enabled reconstruction workstation. Each reconstruction required approximately 10 minutes of computation time. Using only the CT images, images segmentations were performed using the Imalytics software on the MI Labs workstation. Briefly, the skeleton, lungs, and, in some cases, the spleen and mouse bed were segmented using a combination of manual thresholding and free-hand drawing. Afterwards, the mouse kidneys, liver, bladder, and heart were manually segmented and morphologically dilated by 1 voxel. The inferior portions of the liver (near the intestines and stomach) were difficult to differentiate and therefore represent the best approximation of the liver volume. At the time of 15 min, only the mouse body was segmented, which served as the basis for the calibration of the signal for the 6 and 24 h time points. After segmentation, the optical data set was applied as an overlay where metrics can be derived for each organ and mouse. The percent injected dose (%ID) per organ of interest was found by first calculating the average sum total signal from more than 5 mice in each group, which served as the calibration factor S_Tot_. The organ-specific total signal minus autofluorescence was then normalized against S_Tot_, to arrive at the %ID. To test for differences in organ uptake between GAN-fed and NC-fed mice, an ordinary two-way ANOVA was performed where a Bonferroni multiple comparisons correction was then applied. Results were deemed significant at a value p < 0.05. All statistical measurements were performed using GraphPad Prism 8.

### Liver enzyme analysis

Blood samples were collected for enzyme activity measurement in K2-EDTA tubes and kept on ice. The plasma was isolated by spinning the blood tubes at 10,000g x 5 min at 4℃. The resulting supernatant (~400 µL of plasma per mouse) from NC-fed mice (n = 13) and GAN-fed mice (n = 15) was transferred into 2 mL 96-well deep-well plates and stored at -80 ℃. Terminal mouse plasma samples were thawed and diluted with PBS (1:1). Liver and lipid analytes were measured using the Cobas C311 Clinical Analyzer. (Cobas C311 analyzer, Roche Diagnostics).

### Tissue staining and analysis

Following *ex vivo* imaging, the lateral left liver lobe from each animal was harvested. Tissues were fixed in 10% neutral buffered formalin for 24 h, stored in 70% ethanol, then processed and embedded into paraffin. Slides were stained with hematoxylin and eosin (H&E) using standard protocols and picrosirius red staining (PSR) using a Picro-Sirius Red Pint Stain Kit (American MasterTech; StatLab). For PSR staining, the vendor's protocol was modified by pretreating the slides overnight, staining with picrosirius red solution for 30 minutes, and omitting the hematoxylin counterstain. Slides were imaged using a NanoZoomer 2.0HT Slide Scanner (20X objective; Hamamatsu Photonics). Tissues were evaluated for NAS Activity Score, steatosis, lobular inflammation, and hepatocyte ballooning by a blinded histopathologist based on previously described criteria 35. Collagen content was quantified using whole-slide image analysis software (ORBIT Image Analysis; Idorsia Pharmaceuticals Ltd) and was expressed as the percentage of positive PSR-stained area over the total area of measured tissue 36. Large vasculature and empty spaces (primarily spaces of lipid droplets in GAN mice) were excluded from image analysis.

The expression of COL-1 in HFCDA-fed mice livers was quantitatively determined by immunohistochemical (IHC) analysis. Briefly, liver sections (4 µm) were deparaffinized in xylene, immersed in decreasing concentrations of ethanol, and rehydrated in water. The sections were pre-treated using heat mediated antigen retrieval with citrate buffer (pH 6.0) or Tris-EDTA buffer (pH 9.0) for 15 min. Following blocking endogenous peroxidase activity with 3% H_2_O_2_ for 15 min, the sections were further blocked with Animal-Free Blocking Solution (REF 15019L, Cell Signaling) for 20 min at room temperature. The sections were then incubated with primary Anti-COL-1 antibody (REF ab270993; Abcam) for 1 h at room temperature. After several rinses in TBST, they were incubated with the Polymer Detection System (REF MP-7451; Vector Laboratories) for 30 min. The bound peroxidase was visualized by incubating the sections with ImmPACT DAB Peroxidase (HRP) Substrate (REF SK-4105; Vector Laboratories). The slides were counterstained with Modified Mayer's Hematoxylin (REF HXMMHPT; StatLab). Whole slide digital images were captured using a NanoZoomer 2.0HT slide scanner (20X objective; Hamamatsu Photonics). The IHC-positive stained area was quantified using a positive stain measurement algorithm, expressed as a percentage of the positive stained area over the total measured area.

## Results and Discussion

### Synthesis and *in vitro* characterization of affibody fluorescent probes

**Figure 1:**
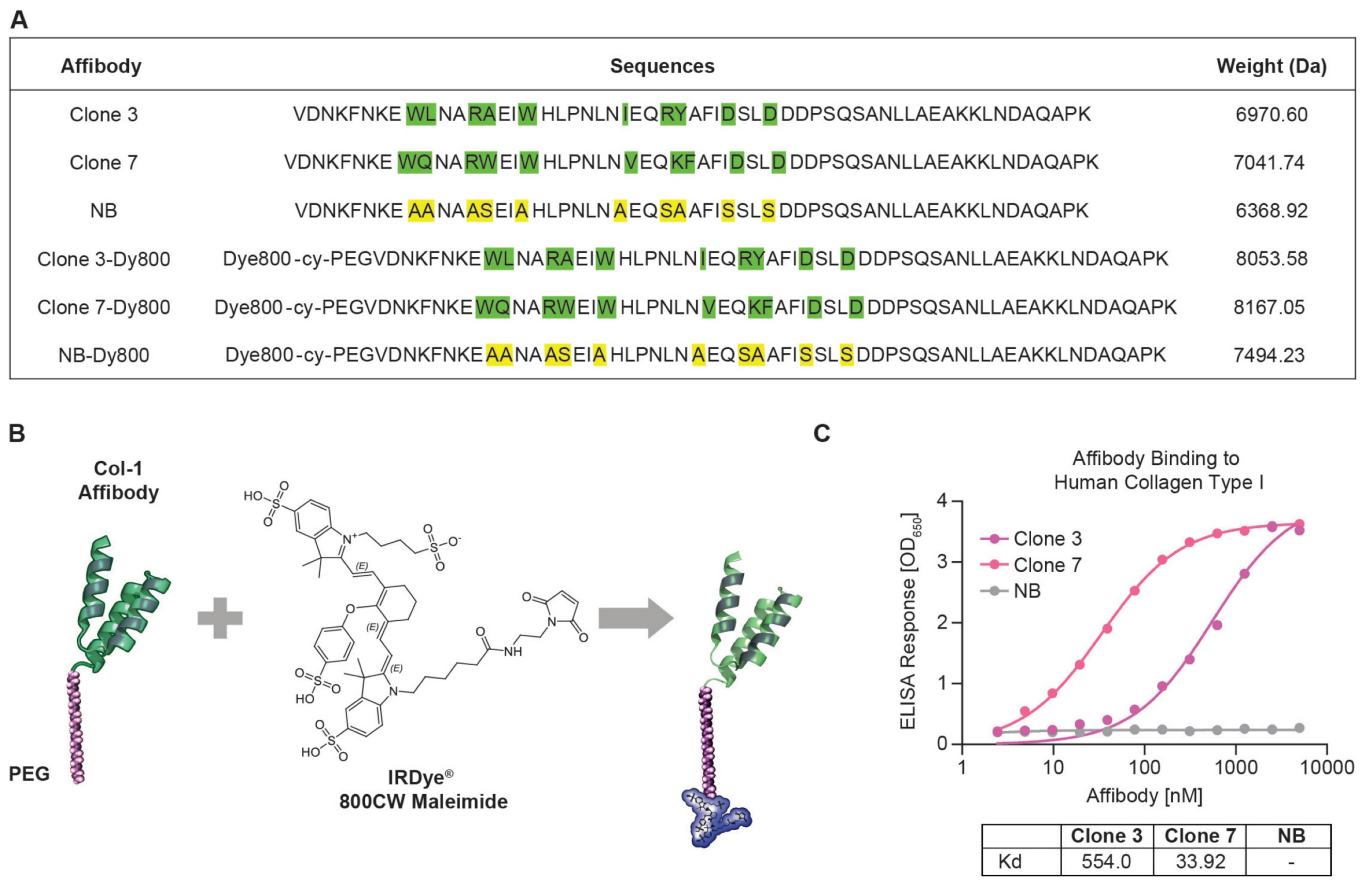
** Characterization of affibody probes and their binding affinity to human COL-1.** (A) Amino acid sequences of Clone 3, Clone 7, NB and their dye conjugated structures, with highlighted binding sites and their molecular masses values, confirming successful synthesis and conjugation of the affibodies with the fluorophore. (B) structural representation of the affibody conjugated to a PEG linker and Dye 800 maleimide. (C) ELISA binding assay for the affinity of the Clone 3, Clone 7 and NB affibodies against human COL-1. NB affibody showed no binding to human COL-1, while Clone 7 demonstrated >15 times higher binding than Clone 3.

Utilizing phage display, we panned a diverse affibody library based on the Z-domain of Staphylococcus protein A and isolated phages with strong human COL-1 binding. We selected seven binders for synthesis and measured their binding affinities to human COL-1 using ELISA. The binding results showed that Clone 3 and Clone 7 exhibited the highest relative binding affinities, with dissociation constant (Kd) values of 554.0 nM and 33.92 nM, respectively (Figure 1A and 1C). A scrambled version of the COL-1-binding affibodies was also engineered as a negative control, demonstrating no binding to COL-1. This variant is structurally identical to the COL-1 binding affibodies, except that the residues in the binding region were substituted with alanines and serines (Figure 1A, Clone NB). All peptides were synthesized via solid-phase chemistry. To improve solubility and stability, a short PEG spacer was introduced at the N-terminus of the affibody (Figure 1B) 37. A cysteine residue was incorporated into the PEG linker to enable site-specific conjugation with a maleimide-functionalized Dy800 dye (Scheme S1). Mass spectrometry data confirmed successful synthesis and conjugation with >90% purity (Figures S1-S6). After synthesis, a short PEG spacer was introduced at the N-terminus of the affibody to improve its solubility and stability, reduce nonspecific uptake and off-target toxicity, and minimize immunogenicity 37.

### *In vivo* optical imaging studies of Clone 3-Dy800

#### Selection of optimal dosage and imaging time point for Clone 3-Dye800 liver fluorescence imaging

Due to the novelty of the COL-1 affibody probes, we conducted a small pilot study to optimize the dosage and imaging time points through *in vivo* 2D FLI in the HFCDA-fed MASH animals and their age-matched NC-fed mice. We selected the Clone 3-Dy800 as our first generated probe and evaluated it for *in vivo* imaging. To determine the optimal dosage of Clone 3-Dy800, we used HFCDA-fed and NC-fed mice (n = 4 each group), injected them with 0.375, 0.75, 1.5, and 2.3 mg/kg (approximately 10, 20, 40, and 60 µg of affibody probes) intravenously and monitored the probe biodistribution at different time points up to 72 h- post-injection. Results showed that the probe was cleared in both HFCDA-fed and NC-fed mice by 72 h across all the dosages used, with more rapid clearance observed in NC-fed mice compared to HFCDA-fed mice. At 24 h, with the highest dosage of 60 µg, the optical signal remained elevated in NC-fed mice, particularly in the abdominal area, suggesting non-specific accumulation in other organs that were not observed at lower dosages. The 40 µg (1.5 mg/kg) dosage provided the highest target-to-background ratio for detecting the probe uptake in the liver. At this dosage, we observed the highest difference in signal intensity between NC-fed and HFCDA-fed mice across all time points. In contrast, at the lower dosages (10 and 20 µg), the data showed a smaller difference between the two groups, reinforcing that 40 µg was more suitable for liver imaging (Figure S7A-B).

After determining the optimal dosage of 40 µg (1.5 mg/kg), we injected HFCDA-fed and NC-fed mice with this dosage and imaged them for liver signal intensity at different time points (0, 2, 4, 6, 8, and 24 h) to identify the optimal imaging time post-injection. We did not extend the study beyond 24 h, because the dosage study showed probe clearance from the body at later time points. Liver signal intensity was significantly higher in HFCDA-fed mice compared to NC-fed mice at 6 h (2.5-fold increase), 8 h (2.0-fold increase), and 24 h (4.5-fold increase) post-injection (p < 0.02), while no significant difference was observed at earlier time points. Thus, an effective imaging window between 6 to 24 h post-injection was determined. (Figure S7C).

#### μCT-FLT imaging of Clone 3-Dy800 liver uptake in GAN mice

**Figure 2:**
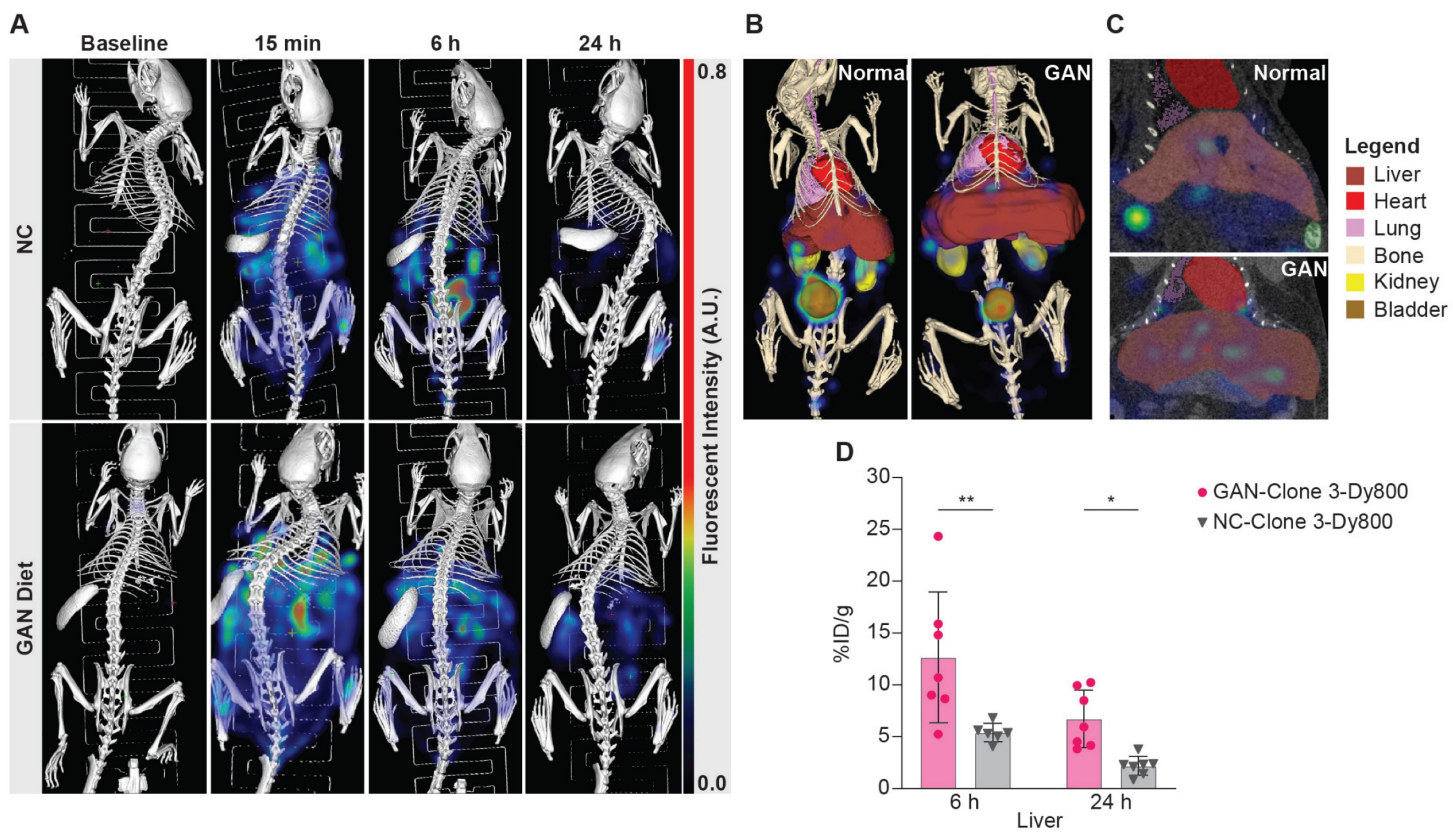
**Representative segmentations and quantification.** (A) 3D FLI images at before and 6hr and 24 h post probe injection. (B) segmented 3D composite views of the NC-fed and GAN-fed at 6h post probe injection, where segmented organs are color-coded (bladder: brown, heart: red, kidneys: yellow, liver: dark red, lungs: purple). (C) Coronal slice view demonstrating liver uptake. (D) Percentage of injected probe (%ID) normalized by body weight calculated from GAN-fed (n = 14) and NC-fed (n = 13) mice at 6 and 24 hs (*P < 0.03 and **P < 0.004). Elevated probe uptake can be seen in the liver at both time points. Note: The small imbalance in group size was due to the loss of one mouse in the NC group during the study period, and not by design. We confirmed that statistical analyses (ANOVA) remain robust with minimal imbalance.

After identifying the optimal Clone 3-Dy800 dosage and imaging time point based on 2D imaging, we performed 3D μCT-FLT (MiLab system) *in vivo* imaging to assess the region-specificity and sensitivity of the imaging probe. We selected a total of 28 mice and divided them equally to feed with GAN diet or NC for 31 weeks. We further divided each group of 14 mice into two subgroups of 7 mice each that received the Clone 3-Dy800 binding probe (1.5 mg/kg), where both groups were imaged *in vivo* prior to probe injection (baseline), at 15 min (right after injection), whereas at a different terminal time point that was set to 6 h in one subgroup and 24 h in the other subgroup, post probe injection (Scheme 1A). The 15 min time point was chosen as the first imaging time point immediately after probe injection, taking into account the time needed to anesthetize the animal and position it for imaging. This early time point was selected to ensure that the full injected dose of the probe remained within the body, with minimal clearance, allowing us to consider this time point as representing 100% of the injected dose (ID). By using this data as a reference, we calculated the percentage of the injected dose per organ (%ID) for all following time points 38. The imaging data showed that Clone 3-Dy800 was dispersed throughout the body at 15 min post-injection, and compared to NC-fed mice, a significantly higher liver uptake in GAN-fed mice was found at 6 h. At 24 h, fluorescence signal decreased in both GAN-fed and NC-fed groups but remained higher in GAN-fed mice (Figure 2A). To quantify the fluorescence signal from the liver, we performed 3D segmentation by outlining the liver with scribbles using Imalytics software, as described in the experimental section and the result was shown in Figure 2B and C (liver is color-coded dark red). Other organs, including the lungs, heart, spleen, kidneys, and bladder, were also segmented using the same method as shown with different color codes in Figure 2B. We then calculated the percentage of injected probe (%ID) normalized by body weight (g) at 6 h and 24 h post-injection. As shown in Figure 2D, the fluorescent signal was significantly higher in the liver of GAN-fed mice at 6 h (%ID/g = 13.8 ± 6.2, n = 7) and 24 h (%ID/g = 6.7 ± 2.5, n = 7), compared to those of fed with NC diet 6 h (%ID/g =5.7 ± 0.8, n = 6) and 24 h (%ID/g = 2.2 ± 0.8, n = 7).

**Figure 3:**
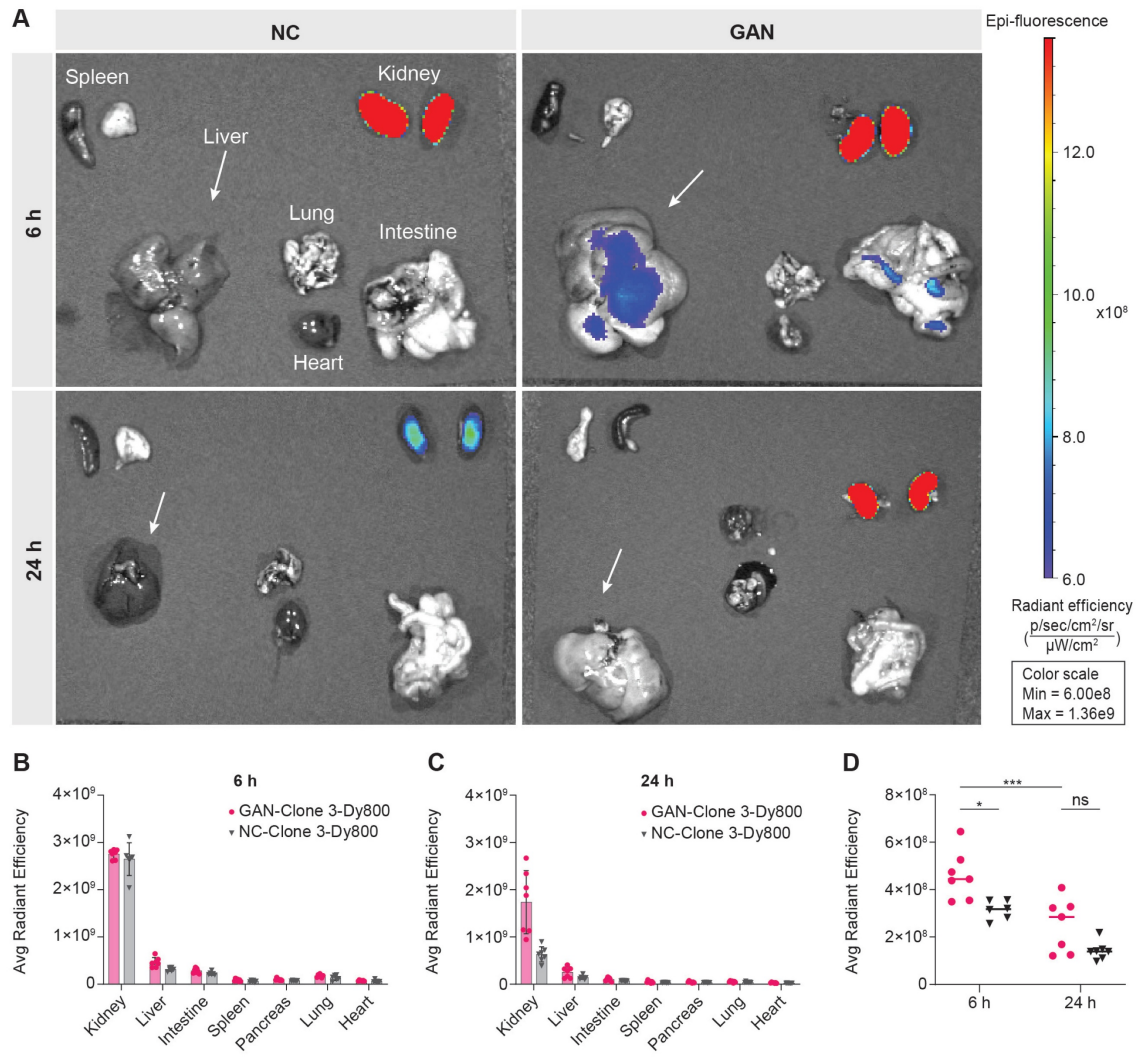
***Ex vivo* biodistribution of Clone 3-Dy800 in major organs.** (A) *Ex vivo* imaging data of the isolated major organs from NC- and GAN-fed mice at 6 h and 24 h post Clone 3-Dy800 injection. (B) Quantitative analysis of the fluorescence signal of the major organs of mice at 6 h post Clone 3-Dy800 injection. (C) Quantitative analysis of the fluorescence signal of the major organs of mice at 24 h post Clone 3-Dy800 injection. (D) Liver signal intensity comparison between NC- and GAN-fed mice at 6 h and 24 h post Clone 3-Dy800 injection (*P < 0.02 and ***P = 0.0004).

Following *in vivo* imaging, we euthanized the mice at 6 h and 24 h post probe injection and collected their major organs for *ex vivo* 2D optical imaging, to corroborate the probe uptake observed from *in vivo* 3D optical imaging. As illustrated in Figure 3A, a significantly higher signal was detected in the kidneys of both GAN-fed and NC-fed mice, indicating renal clearance of the probe. The signal remained elevated at 24 h post-injection, particularly in GAN-fed mice. This difference in renal fluorescence likely reflects altered probe clearance dynamics, with NC mice exhibiting a faster clearance rate. Minimal or no signal was observed in the other major organs at both time points (Figures 3A-C). The liver signal was higher in GAN-fed mice than in NC-fed mice at both 6 and 24 h, consistent with the *in vivo* imaging data (Figure 3D).

**Figure 4:**
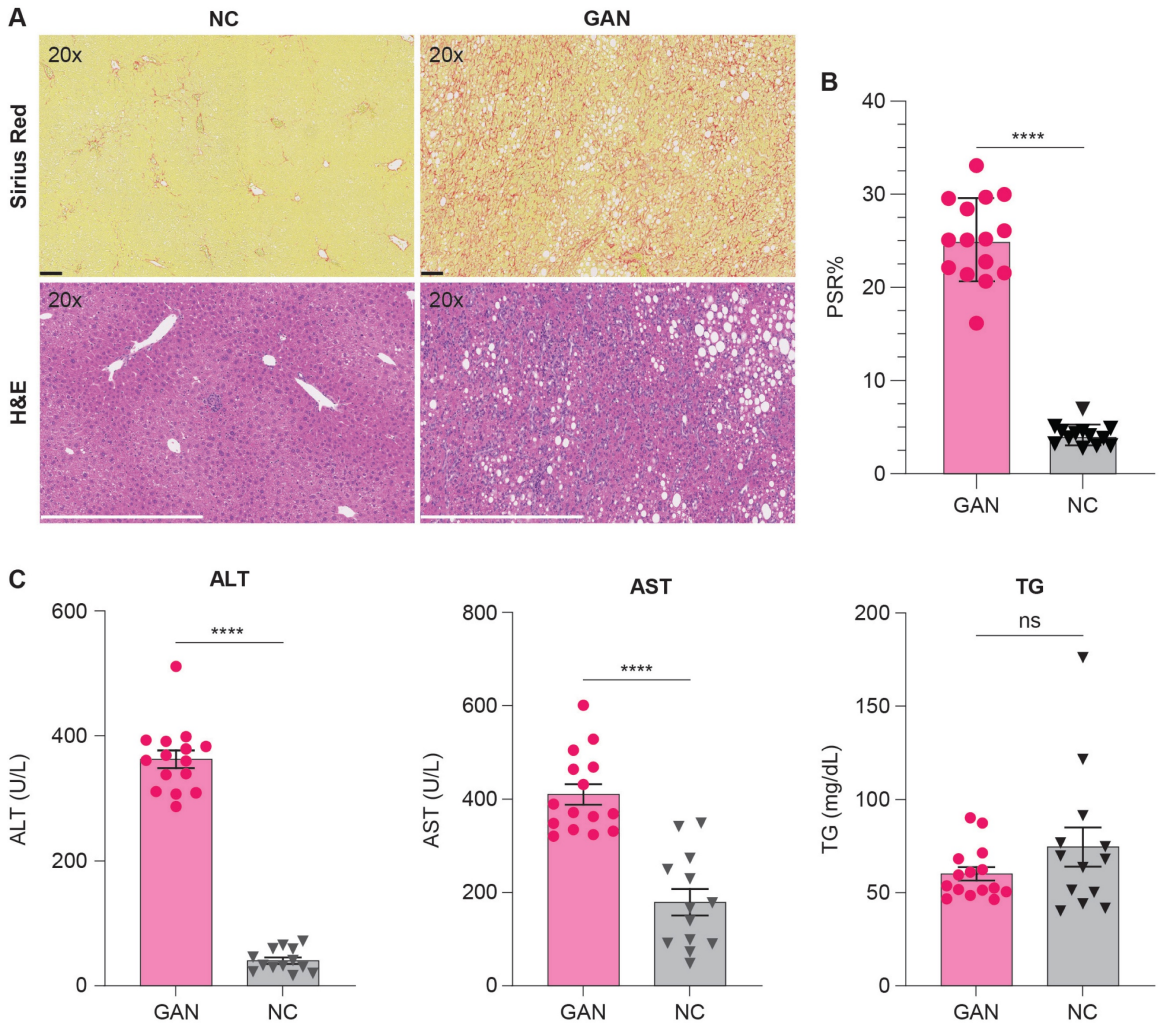
**Histological and biochemical assessment of liver fibrosis and metabolic changes in NC- and GAN-Fed mice.** (A) Representative images of liver tissue from mice fed either NC or GAN diets, with Picro-Sirius red (PSR) staining (top row) to assess fibrosis and hematoxylin and eosin (H&E) staining (bottom row) for general tissue architecture. (B) Morphometric analysis of PSR-stained sections from each group (n = 15/group), with results expressed as the percentage of tissue area positive for PSR3 staining, highlighting the degree of fibrosis (****p < 0.0001). (C) Serum levels of triglycerides, aspartate aminotransferase (AST), and alanine aminotransferase (ALT) were measured to assess diet-induced metabolic changes (*****p < 0.0001).

The histopathological analysis was performed using Hematoxylin and Eosin (H&E) and Picro-Sirius Red (PSR) stains to evaluate liver tissue and collagen content in GAN-fed and NC-fed mice. Left lateral liver lobes were collected, preserved, and analyzed by a blinded histopathologist for examination of steatosis, lobular inflammation, hepatocyte ballooning, and the type of steatosis. The GAN-fed mice were significantly heavier than the NC-fed mice and developed moderate to severe steatosis and inflammation with elevated liver enzymes, alanine aminotransferase (ALT) and aspartate aminotransferase (AST) levels, and triglycerides (TG) (Table 1, Figure 4C). Fibrosis ranged from moderate to severe in GAN-fed mice, with PSR staining indicating lobular and periportal collagen deposition. Collagen content was quantified using ORBIT image analysis software, which calculated the percentage of positively stained PSR areas relative to the total tissue area, as shown in Figure 4B. The GAN-fed mice had a significant increase in PSR staining compared to the NC-fed mice (p ≤ 0.0001; Figure 4B). Together, these results confirmed the presence of fibrotic liver in this GAN-fed mouse model (Figure 4C).

Additional pathological characterization was performed to further show the association between increased COL-1 expression and fibrosis severity in the GAN model. Quantitative image analysis of COL-1 immunofluorescence staining was performed on liver tissues from GAN-fed and healthy NC-fed mice. As summarized in table S1, COL-1 expression increases with disease severity, correlating with higher NAS scores. Specifically, GAN mice with NAS scores of 7 exhibited %COL-1 area values ranging from 3.12-4.40%, whereas NC mice showed significantly lower COL-1 levels (0.62-0.86%) and lower NAS scores (3-6).

### COL-1 binding and non-binding probes liver uptake in HFCDA mice

The HFCDA is a commonly used diet to induce liver fibrosis in animal models of MASH, making it a valuable tool for testing new therapies with good translation to human disease. Unlike GAN-fed mice, HFCDA shows faster disease progression (8 weeks in C57BL/6J mice for HFCDA vs. over 28 weeks for GAN), make this model more suitable for testing the optimized variant of the COL-1 probe. Using the HFCDA model, we evaluated the effect of binding affinity of two probes, Clone 3-Dy800 and Clone 7-Dy800 and assessed their specificities by comparing them to a non-binding probe *in vivo*. A total of 26 C57BL/6J mice were divided into two groups: 11 on the NC diet and 15 on the HFCDA diet. There were no differences in body weight between mice on NC or HFCDA diet (Table 2). The mice received intravenous injections of different probes: Clone 3-Dy800 (HFCDA = 5, NC = 4), Clone 7-Dy800 (HFCDA = 5, NC = 4), and NB-Dy800 (HFCDA = 5, NC = 3), each at a dose of 1.5 mg/kg. The reduced number of mice in the NB-Dy800 NC group (NC = 3) was due to a technical issue encountered during the study: a few mice in this group developed unexpected skin pigmentation, which interfered with optical imaging by blocking or distorting the near-infrared signal. As a result, we had to exclude these animals from the data analysis, to ensure data consistency and comparability across groups.

**Figure 5:**
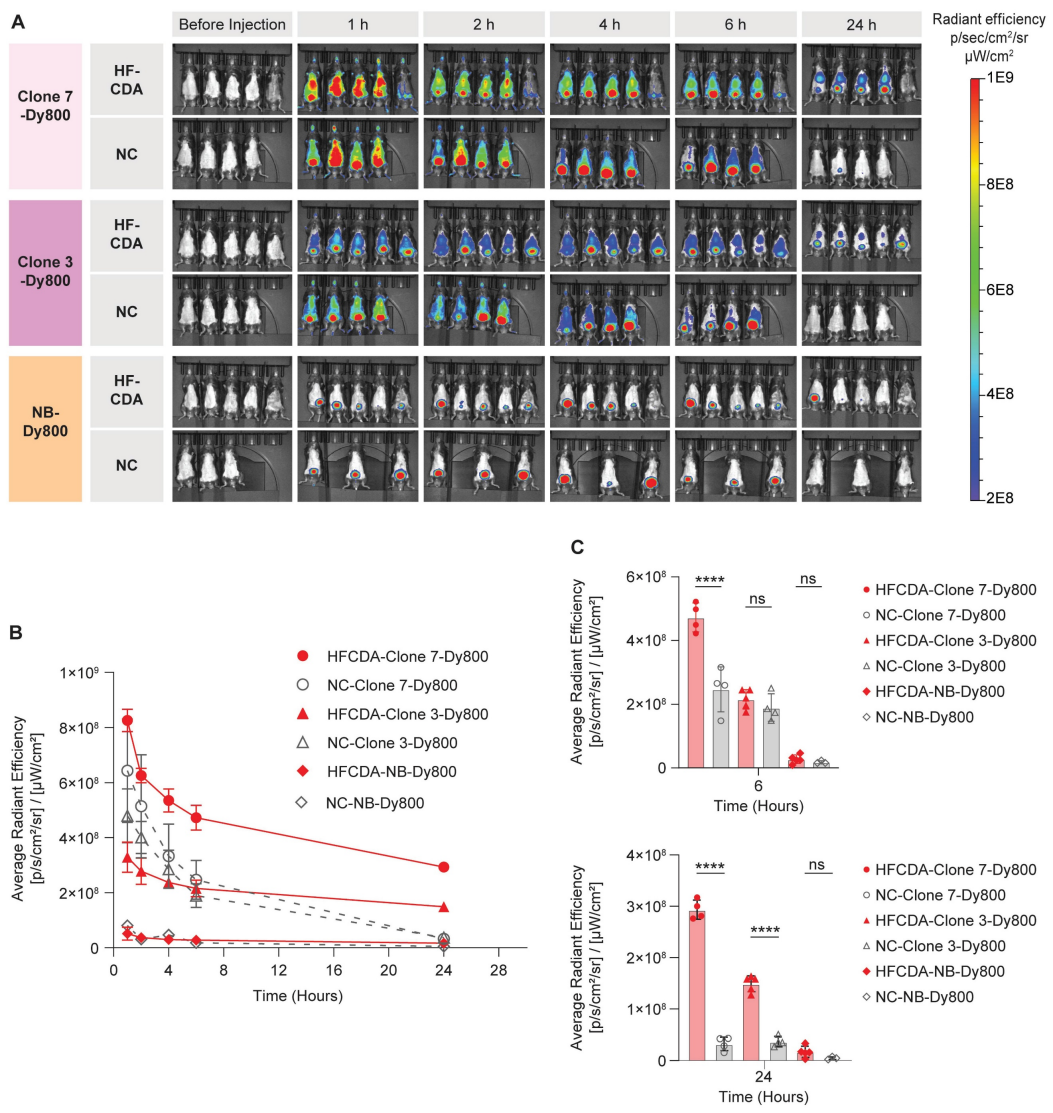
**Representative *in vivo* images and temporal analysis of probe uptake in the livers of mice fed with NC and HFCDA.** (A) *In vivo* 2D optical images of probe distribution in mice. (B) Quantification of signal intensity before and after intravenous injection of Clone 7-Dy800, Clone 3-Dy800, and NB-Dy800 probes, measured at 1, 2, 4, 6, and 24 h post-injection. (C) Comparison of liver uptake signal intensity among different groups at 6hr and 24 h. Higher accumulation in collagen-rich fibrotic livers in HFCDA-fed mice was observed in both Clone 3-Dy800 and Clone 7-Dy800 groups. Results are expressed as mean ± SD (****P < 0.0001)*.*

Biodistribution and probe accumulation in the liver were monitored at various time points using the IVIS Spectrum imaging system. All three probes showed rapid clearance from both NC and HFCDA groups within 24 h, with a faster clearance in NC-fed mice (Figure 5A). A significantly higher signal in the bladder indicated predominant renal clearance. We quantified average fluorescence signal intensity from the liver by drawing an ROI around the chest area for all mice (Figure 5B and Figure S8). The quantified results showed significantly higher signal intensity in livers in HFCDA-fed mice compared to NC-fed mice for both Clone 3-Dy800 and Clone 7-Dy800. The difference in signal intensity between HFCDA-fed and NC-fed mice increased over time for both probes (Figure 5C). Specifically, the liver of HFCDA-fed mice receiving Clone 7-Dy800 showed around two-fold higher signal compared to Clone 3-Dy800, demonstrating higher probe accumulation, likely due to the increased COL-1 binding affinity. Additionally, no signal was observed from the NB-Dy800 probe in the liver of either HFCDA-fed or NC-fed mice.

**Figure 6:**
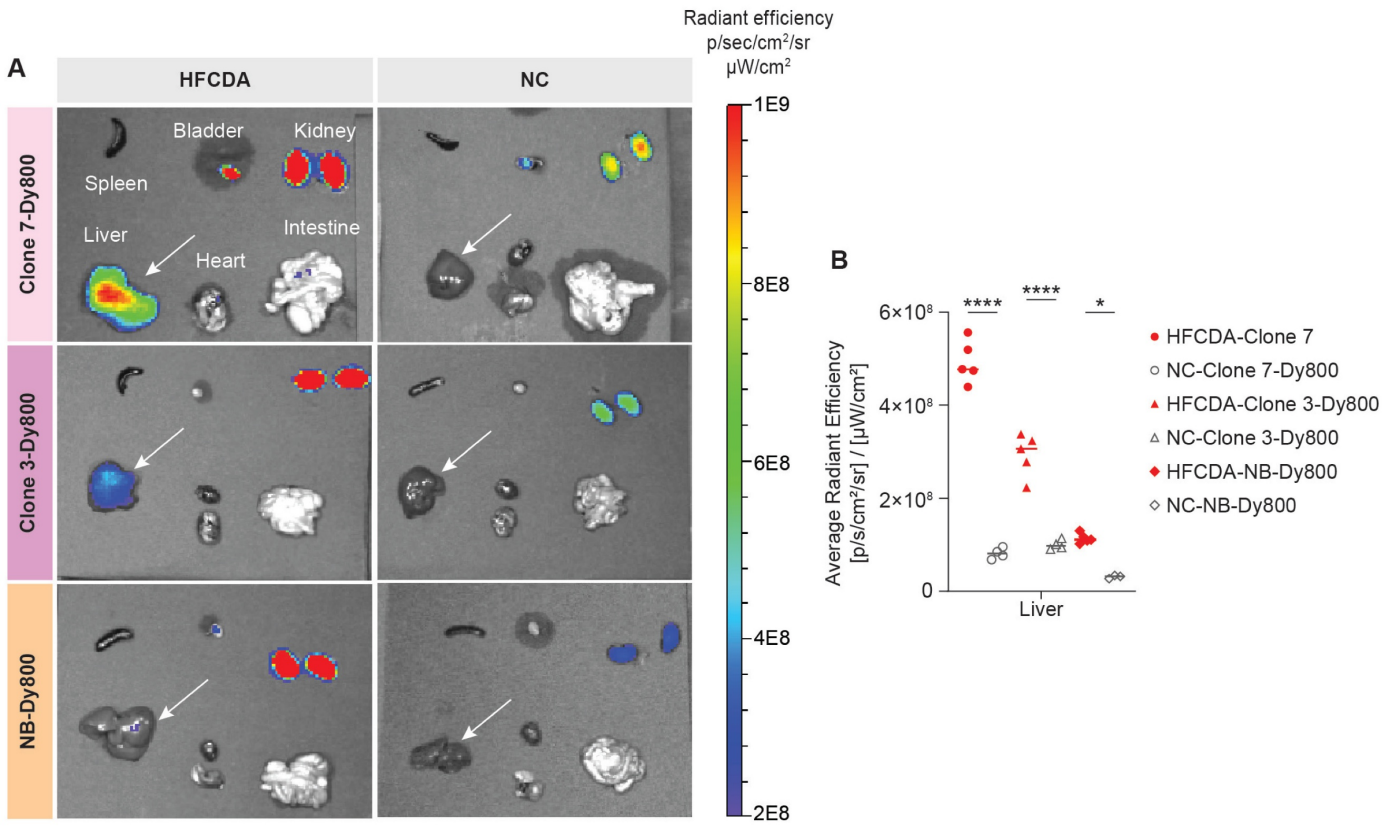
***Ex vivo* biodistribution of affibody probes in major organs.** (A) *Ex vivo* optical imaging of various organs. (B) Quantified fluorescence intensity of Clone 7-Dy800, Clone 3-Dy800, and NB-Dy800 probes, measured at 24 h post-injection. Liver accumulation was the highest in the HFCDA-fed mice: Clone 7-Dy800 > Clone 3-Dy800 > NB-Dy800 in the HFCDA group (*p < 0.02, ****p < 0.0001).

We validated the *in vivo* imaging data by conducting *ex vivo* imaging of major organs collected from both HFCDA-fed and NC-fed mice at 24 h post-probe injection (Figure S9). A higher signal was detected in the fibrotic liver with both, Clone 7-Dy800 and Clone 3-Dy800, confirming our *in vivo* imaging results (Figures 6 A and B). In HFCDA-fed mice, the liver signal observed from the Clone 7-Dy800 group was 1.68 times higher than that of the Clone 3-Dy800 group and 4.33 times higher than the NB-Dy800 group. In contrast, healthy mice showed low liver signals for all three probes. The slightly higher signal in the NB-Dy800 compared to the HFCDA-fed mice is likely due to physiological differences between fibrotic and healthy mice, rather than specific probe binding. Liver fibrosis is associated not only with collagen deposition but also with disrupted architecture, inflammation, and impaired metabolic and clearance function. These changes, including downregulation of key drug-metabolizing enzymes, can reduce probe clearance and lead to increased background retention in fibrotic livers 39.

As a result, even the non-binding probe (NB-Dy800) may show modestly higher retention in fibrotic livers compared to normal ones. However, it is important to note that the signal from NB-Dy800 remains significantly lower than that of the COL1-specific binding clones (Clone 3 and Clone 7), and it also clears more rapidly over time. These differences support the conclusion that the increased retention and signal observed with Clone 3 and Clone 7 are primarily due to specific collagen binding, rather than nonspecific accumulation.

**Figure 7:**
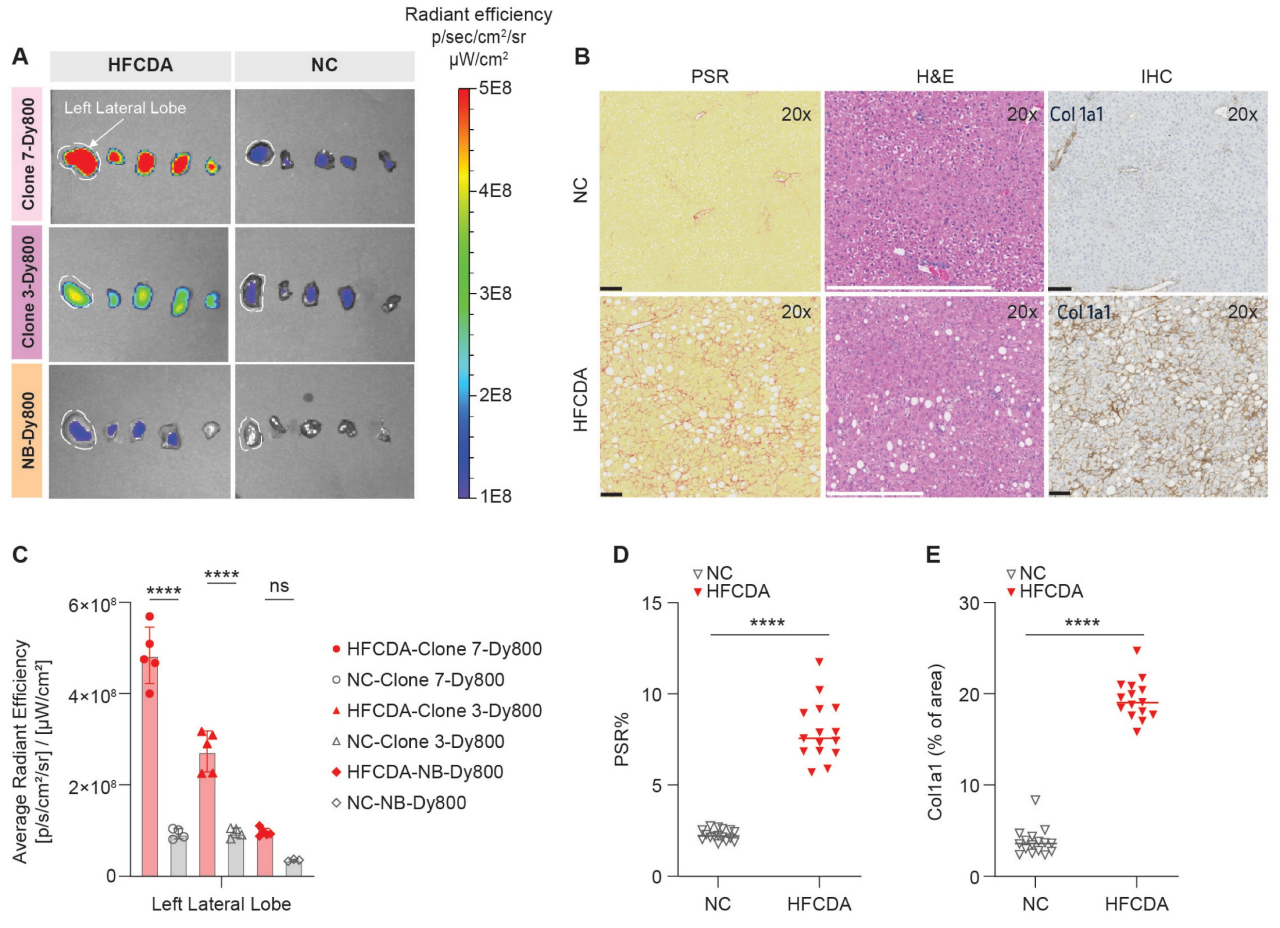
***Ex vivo* imaging and histological assessment of liver fibrosis in mice fed NC and HFCDA diet. (**A) Representative images of liver lobes (left to right: left lateral, left medial, right medial, right lateral, and caudate, from left to right) in mice fed NC or HFCDA diets, 6 h post-injection of Clone 7-Dy800, Clone 3-Dy800, and NB-Dy800 probes. (B) Picro-Sirius red (PSR) staining, H&E, and COL-1 staining for NC diet (top row) and HFCDA diet (bottom row) in liver left lateral lobe. (C) Quantified optical intensity from the liver left lateral lobe. (D) Morphometric analysis of PSR stained sections from each group, with results expressed as the percentage of tissue area positive for PSR staining, showing the degree of fibrosis (****p < 0.0001). (E) Quantification of COL-1 IHC-positive stained areas using a positive stain measurement algorithm, with results expressed as the percentage of positively stained area relative to the total measured area (****P < 0.0001). For all of the experiments shown in this Figure: n = 5 for the HFCDA mice/group, n = 4 for the NC/group.

**Figure 8:**
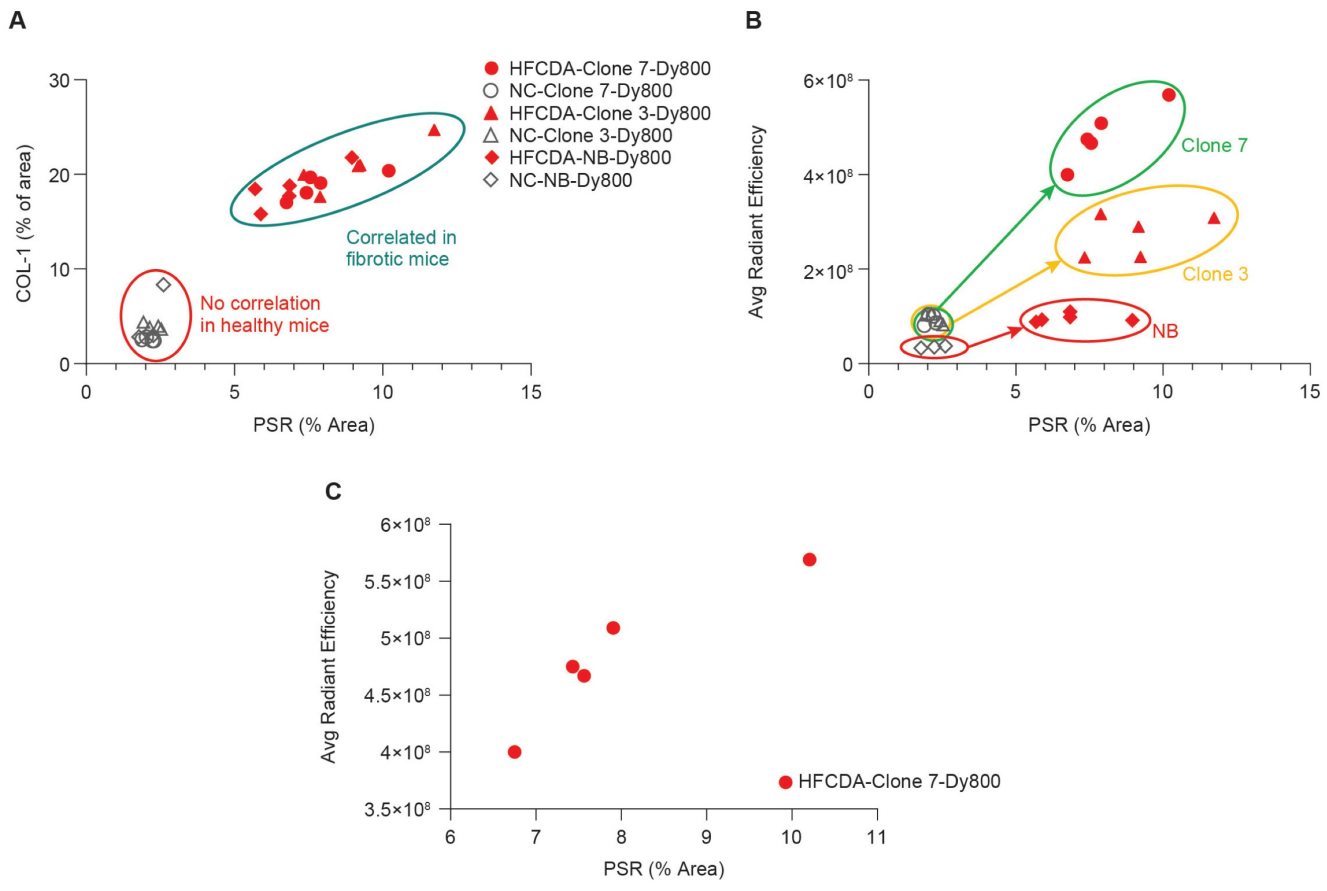
**Correlation of liver fibrosis and probe signal intensity in NC- and HFCDA-fed Mice.** (A) Correlation between PSR staining and COL-1 expression in HFCDA-fed and NC-fed mice, highlighting the relationship between liver fibrosis and collagen deposition. (B) Correlation between average signal intensity from the left lateral liver lobes imaged with different probes (Clone 7-Dy800, Clone 3-Dy800, and NB-Dy800). (C) Correlation between liver signal intensity from the Clone 7-Dy800 and PSR values (r^2^ = 0.86). For all the experiments shown in this Figure: HFCDA-fed mice = 5/group, NC-fed mice = 4/group.

The left lateral liver lobes from HFCDA and NC mice were analyzed for probe accumulation and signal intensity (Figure 7A-B), using the same lobes as those assessed for histology. The HE results showed that the HFCDA diet induced moderate steatosis (from micro- to macrovesicular) and lobular inflammation (characterized by infiltration of mononuclear cells and neutrophils), with marked fibrosis (primarily in the perisinusoidal region) (Figure 7B and Table 2). In addition, these mice presented with varying levels of pigment-laden foam cells, which are mini clusters of mononuclear cells surrounded by lipidic cells with a visible “foamy” pigmentation. NC-fed mice exhibited minimal changes in fat deposition, immune cell infiltration, and collagen deposition, indicating no significance in liver pathology. This observation was confirmed using PSR staining and COL-1 IHC, indicating mice on the HFCDA diet had significantly increased levels of COL-1 expression in the liver compared to NC-fed mice (Figure 7D-E). A strong correlation was observed between COL-1 expression and PSR quantification (Figure 8B), confirming the association between increased COL-1 levels and fibrosis severity in the HFCDA model.

The correlation between histology and *ex vivo* imaging data of the liver left lateral lobe confirmed that both probes effectively distinguished between fibrotic and non-fibrotic livers (Figure 8B). Specifically, a strong correlation was observed between liver signal intensity from the Clone 7-Dy800 and liver PSR values (r^2^ = 0.86, Figure 8C), demonstrating the ability of affibody-based probes with higher binding affinity to non-invasively detect liver fibrosis. To evaluate the relationship between Clone7-Dy800 signal and histological markers of fibrosis, we summarized COL-1 expression, PSR staining, NAS score, and *in vivo* fluorescence signal (average radiant efficiency) in both HFCDA and NC animals (Table S2). As shown, animals with elevated COL-1 and PSR% showed markedly higher probe uptake, supporting the probe's sensitivity to fibrotic ECM remodeling. As shown in Figure S10, a strong positive correlation was observed between probe signal and COL-1 area (R² = 0.77).

Finally, we evaluated the thermal stability of Clone7 and Clone7-Dy800 to support potential clinical transition and long-term storage (Figure S11). Using the Prometheus Panta system, both constructs showed a high melting temperature (T_m_) of ~71 °C. The unfolding and refolding transitions occur at identical temperatures (~71 °C and ~70 °C, respectively), suggesting that the folding process is reversible with minimal hysteresis. This reversible behavior reflects the affibody's ability to maintain its structural integrity under thermal stress, a desirable property for biotechnological and biomedical applications. Furthermore, similar thermal profiles were obtained for Clone 7 and Clone 7-Dy800, confirming that dye labeling does not compromise protein stability. Dynamic light scattering (DLS) analysis confirmed that both Clone7 and Clone7-Dy800 maintained a small, monodisperse size distribution, with no evidence of aggregation. These results demonstrate that Clone7 retains its structural integrity and colloidal stability after labeling, further supporting its suitability for *in vivo* and diagnostic applications.

In summary, COL-1 targeting affibody probe exhibits molecular specificity, offering potential use for detecting disease-related changes beyond structural alterations observable in other established noninvasive modalities such as vibration-controlled transient elastography (VCTE) and MR elastography (MRE) are widely used to assess liver stiffness, which correlates with fibrosis. However, these techniques are primarily sensitive to mechanical tissue changes and may lack the specificity to distinguish between MASH and other causes of stiffness. For instance, VCTE is limited in detecting early stages of the diseases and can be less accurate in obese patients, while MRE is more robust but costly and technically demanding 40. In contrast, our molecular imaging probe targets disease-associated molecular features, potentially enabling earlier detection of MASH, including stages prior advanced fibrosis.

Our study has several limitations that should be examined further. First, optical imaging is not translatable to the clinic. We primarily used this method for initial characterization of candidate probes and selected those with higher sensitivity and specificity to detect fibrotic livers. To this end, one can conjugate the affibody probes with radioisotopes or magnetic nanoparticles instead for PET or MR imaging, and this modification can be easily applied to the affibody scaffolds and potentially enhance sensitivity and accuracy with clinical translation. Second, additional studies to evaluate the feasibility of using these imaging probes for monitoring disease progression and responses to novel therapeutics remain to be explored. Finally, besides the initial assessment of the specificity to COL-1 by comparing the liver signal intensity between COL-1 binding and non-binding probes, a blocking study with unconjugated affibody can be more effective in confirming the binding specificity to COL-1.

## Conclusions

We have developed *in vivo* imaging probes using affibody molecules that bind to COL-1, a marker of liver fibrosis, conjugated with the fluorescent Dy800 dye. We synthesized multiple variants and demonstrated that the probes can be engineered to target COL-1 with high binding affinity (in the nanomolar range) and specificity. Compared to healthy controls, elevated liver signal intensity was observed in diseased animal models of MASH, suggesting that affibody-based imaging probes are a promising alternative to liver biopsy for the diagnosing and longitudinal monitoring of liver fibrosis.

## Supplementary Material

Supplementary methods, figures and tables.

## Figures and Tables

**Table 1:** Effect of diet on body weight and indices of liver damage in mice on NC and GAN diets (40% fat kcal, 22% fructose, and 2% cholesterol).

	NC (n = 13)	GAN (n = 14)	*p-value*
**Body weight (g)**	31.6 ± 2.47	41.3 ± 5.819	0.0004
**NAS**	2.23 ± 1.69	6.93 ± 0.62	<0.0001
**Steatosis**	0.92 ± 1.26	2.93 ± 0.27	<0.0001
**Lobular Inflammation**	0.92 ± 0.49	2.07 ± 0.27	<0.0001
**Hepatocyte Ballooning**	0.38 ± 0.65	1.93 ± 0.27	<0.0001

Values represent mean ± SD (n = 13-14). NAS = NAFLD activity score.

**Table 2:** Effect of diet on body weight and indices of liver damage in mice on NC and HFCDA diets.

	NC (n = 11)	HFCDA (n = 15)	*p-value*
**Body weight (g)**	26 ± 1.6	26 ± 1.5	0.3362
**NAS**	0.55 ± 0.93	4.7 ± 0.7	<0.0001
**Steatosis**	0.18 ± 0.4	1.9 ± 0.26	<0.0001
**Lobular inflammation**	0.18 ± 0.4	2.3 ± 0.46	<0.0001
**Hepatocyte ballooning**	0.18 ± 0.4	0.53 ± 0.52	0.1092

Values represent mean ± SD (n = 11-15). NAS = NAFLD activity score.
