# Supplementary material for: Affibody-based optical imaging probe for noninvasive detection of liver fibrosis
Source: Theranostics. 2026 Jan 1;16(1):1–16. doi: 10.7150/thno.117262 (PMC12665049; doi:10.7150/thno.117262)
Supplement: Supplementary file 1 — Supplementary methods, figures and tables. [file thnov16p0001s1.pdf]

Affibody-based optical imaging probe for noninvasive detection of liver fibrosis

Raana Kashfi Sadabad[1], Paul Stephen Marinec[1], Lawrence Lechuga[1], Robert Thompson[1], Ran Cheng[2], Ashmita Saigal[1], Irodiel Vinales[3], Salomon Martinez[1], Allan Eduardo Solis[1], Emily Melody Tso Newman[1], Una Goncin[1], Christopher Novotny[1], Corey Miller[1], Chih-Liang Chin[1]

[1]Merck & Co., Inc., Rahway, NJ, USA.

[2]Department of Chemistry, Boston University, Boston, MA 02215, USA.

[3]Department of Chemistry and Biochemistry, University of Texas at El Paso, El Paso, Texas 79968, USA.

Corresponding authors:

Chih-Liang Chin, Ph.D., Email: chih-liang.chin@merck.com

Raana Kashfi Sadabad, PhD., Email: kashfisa@merck.com

Fmoc-Strategy Solid phase peptide synthesis (SPPS) General

Clone 7 synthesis:

Resin loading: 0.31 g of Rink Amide MBHA resin (0.1 mmol, 0.33 mmol/g) was transferred to a 20 mL reaction vessel. The resin was swollen with 30 mL of DMF (2hrs).

Coupling of Fmoc-Lys(Boc)-OH: Deprotection of the Fmoc group was accomplished with 20 mL of 20% 4-methylpiperidine in DMF to the swollen resin for 30 min. After deprotection the resin was washed with 30 mL of DMF (5 x 0.1 min) and followed by addition of Fmoc-Lys(Boc)-OH (0.30 mmol, 140.6 mg, 3.00 equiv.), HBTU (0.285 mmol, 108.3 mg, 2.85 equiv.) and DIEA (0.60 mmol, 0.105 mL, 6.00 equiv.) in DMF (10 mL). After completing the coupling reaction, the resin was washed with 30 mL of DMF (3 x 0.1 min) prior to starting the next deprotection/coupling cycle.

Coupling of Ac: Deprotection of the Fmoc group was accomplished with 20 mL of 20% piperidine in DMF to the swollen resin for 30 min. After deprotection the resin was washed with 30 mL of DMF (5 x 0.1 min) and followed by addition of 10% Ac_2O/5% NMM/85% DMF (20 mL) in resin.

The coupling reaction was mixed for 30 min, filtered. After completing the coupling reaction, the resin was washed with 30 mL of DMF (5 x 0.1 min) and 30 mL of MeOH (3 x 0.1 min), then dried under vacuum.

Cleavage: TFA cleavage and isopropyl ether precipitation: 10 mL of the TFA cleavage cocktail (TFA/water/TIS/3-Mpa: 92.5/2.5/2.5/2.5) was added to the protected resin bound peptide and shaken for 2 hrs. Cold isopropyl ether was added to form a white precipitate which was then pelleted by centrifugation. The supernatant was decanted to waste and two more isopropyl ether washes of the precipitate were performed. The crude linear peptide was dried under a vacuum for 2 hrs. Clone 7 (0.68 g, crude) was obtained in the form of a white solid.

Purification: RP-HPLC purification: Semi-Preparative reverse phase HPLC was performed on a YMC-Actus 10 µm C18 column (30 mm x 250 mm) (GLX-281). Separations were achieved using linear gradients of buffer B in A (Mobile phase A: water containing 0.075% TFA, mobile phase B: Acetonitrile (ACN)), at a flow rate of 20 mL/min (preparative). The Gradient was 16-36-40 min and Retention time was 33 min.

Final lyophilization and analysis: The collected fractions were analyzed by analytical RP-HPLC. COL-1 affibody was obtained in the form of a white solid with a purity of 97% after lyophilization. After purification, 102 mg of peptide was obtained, corresponding to a yield of ~14.2% relative to the theoretical maximum (720.23 mg). Low-resolution LC/MS of purified COL-1 affibody gave 9 charged states of the peptide: $[M+4H]^{4+}$=1761.9; $[M+5H]^{5+}$=1409.4; $[M+6H]^{6+}$=1174.7; $[M+7H]^{7+}$=1007.1; $[M+8H]^{8+}$=881.3; $[M+9H]^{9+}$=783.6; $[M+10H]^{10+}$=705.3; $[M+11H]^{11+}$=641.3; $[M+12H]^{12+}$=587.8. The experimental mass agrees with the theoretical mass of 7041.84 Da [M+1].

COL-1 non-binding affibody molecule synthesis and analysis

Resin loading: 0.78 g of Rink Amide MBHA resin (0.5 mmol, 0.33 mmol/g) was transferred to a 50 mL reaction vessel. The resin was swollen with 50 mL of DMF (2 hrs).

Coupling of Fmoc-Lys(Boc)-OH: Deprotection of the Fmoc group was accomplished with 50 mL of 20% piperidine in DMF to the swollen aldehyde resin for 30 min. After deprotection the resin was washed with 30 mL of DMF (5 x 0.1 min) and followed by addition of Fmoc-Lys(Boc)-OH (1.50

mmol, 702.75 mg, 3.00 equiv.), HBTU (1.425 mmol, 541.5 mg, 2.85 equiv.) and DIEA (3.00 mmol, 0.50 mL, 6.00 equiv.) in DMF (30 mL). After completing the coupling reaction, the resin was washed with 50 mL of DMF (3 x 0.1 min) prior to starting the next deprotection/coupling cycle.

Coupling of Ac: Deprotection of the Fmoc group was accomplished with 20 mL of 20% piperidine in DMF to the swollen resin for 30 min. After deprotection the resin was washed with 50 mL of DMF (5 x 0.1 min) and followed by addition of 10% Ac_2O/5% NMM/85% DMF (20 mL). The coupling reaction was mixed for 30 min, filtered. After completing the coupling reaction, the resin was washed with 50 mL of DMF (5 x 0.1 min) and 30 mL of MeOH (3 x 0.1 min), then dried under vacuum.

Cleavage: TFA cleavage and isopropyl ether precipitation: 40 mL of the cleavage cocktail TFA cleavage cocktail (TFA/water/TIS/3-Mpa: 92.5/2.5/2.5/2.5) was added to the protected resin bound peptide and shaken for 2 hrs. Cold isopropyl ether was added to form a white precipitate that was then pelleted by centrifugation. The supernatant was decanted to waste and two more isopropyl ether washes of the precipitate were performed. The crude linear peptide was dried under a vacuum for 2 hrs. COL-1 non-binding affibody molecule (2.3 g, crude) was obtained in the form of a white solid.

RP-HPLC purification: Semi-Preparative reverse phase HPLC was performed on a Welch Sail-1000, Welch Ultimate® XB-C18, 250*50 mm, 7 µm, 120Å + Welch Xtimate® C18 250*50 mm, 10 µm, 120Å. Separations were achieved using linear gradients of buffer B in A (Mobile phase A: water containing 0.075% TFA, mobile phase B: Acetonitrile (ACN)), at a flow rate of 80 mL/min (preparative). The Gradient was 41-61-40 min and Retention time was 32 min.

Final lyophilization and analysis: The collected fractions were analyzed by analytical RP-HPLC.

COL-1 non-binding affibody was obtained in the form of a white solid with a purity of 93.8% after lyophilization. After purification, 120 mg of peptide was obtained, corresponding to a yield of ~7.3 % relative to the theoretical maximum (0.2574 mmol scale). Low-resolution LC/MS of purified sample gave 6 charged states of the peptide: $[M+4H]^{4+}$=1593.2; $[M+5H]^{5+}$=1275.4; $[M+6H]^{6+}$=1062.5; $[M+7H]^{7+}$=910.9; $[M+8H]^{8+}$=797.1; $[M+9H]^{9+}$=708.4. The experimental mass agrees with the theoretical mass of 6368.92 Da [M+1].

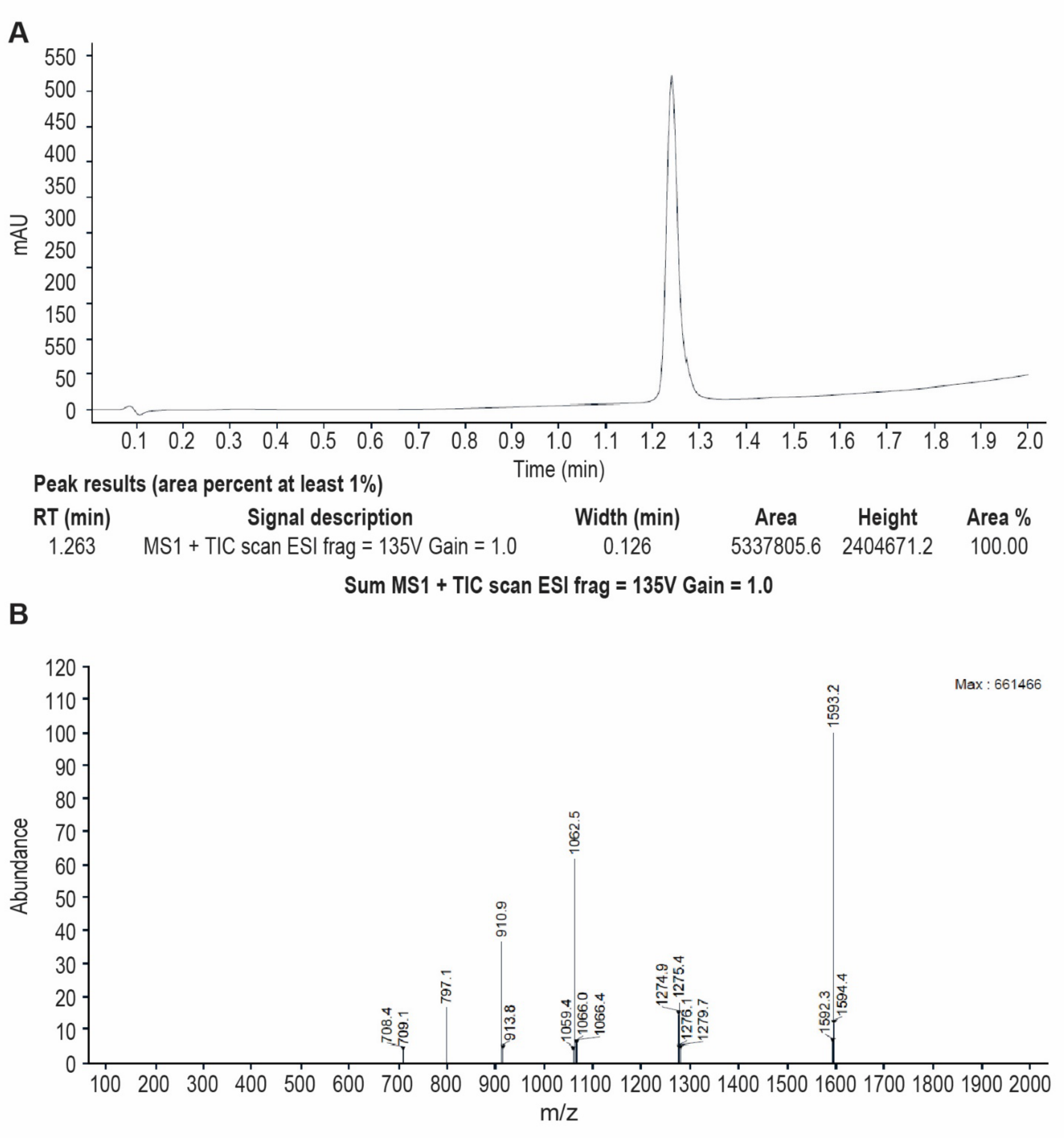

Figure S1. Characterization of NB affibody: (A) UPLC chromatogram. (B) Mass spectrum of the purified peptide. Observed m/z values include: [M+4H]4+=1593.2; [M+5H]5+=1275.4; [M+6H]6+=1062.5; [M+7H]7+=910.9; [M+8H]8+=797.1; [M+9H]9+=708.4. The experimental mass agrees with the theoretical mass of 6368.92 Da [M+1].

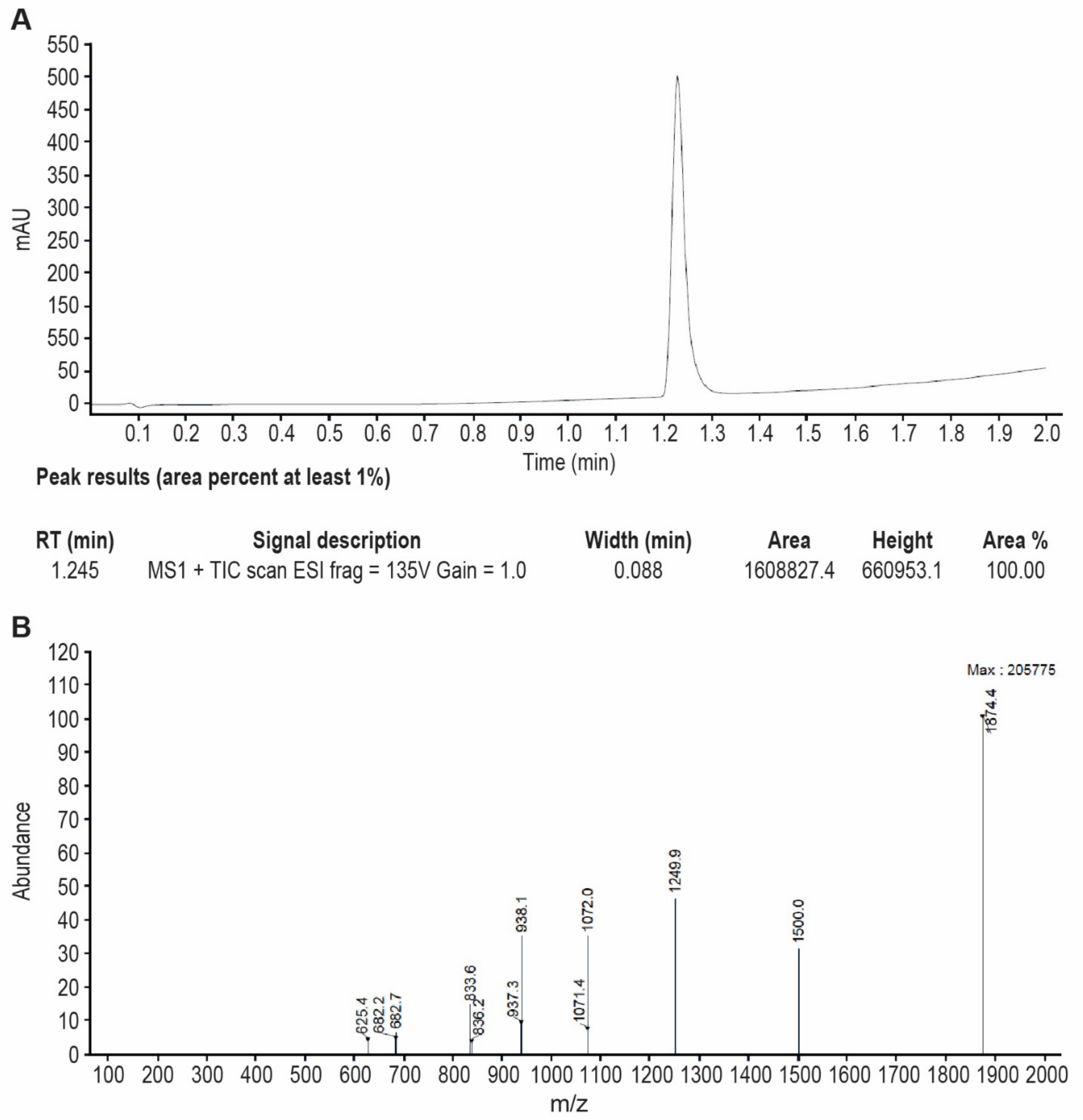

Peak results (area percent at least 1%)

RT (min)	Signal description	Width (min)	Area	Height	Area %
1.245	MS1 + TIC scan ESI frag = 135V Gain = 1.0	0.088	1608827.4	660953.1	100.00

Figure S2. Characterization of NB-Dy800 affibody: (A) UPLC chromatogram. (B) Mass spectrum of the purified peptide. Observed m/z values include: [M+4H]4+=1874.4; [M+5H]5+=1500.0; [M+6H]6+=1249.9; [M+7H]7+=1072.0; [M+8H]8+=938.1; [M+9H]9+=833.6; [M+11H]11+=862.7; [M+12H]12+=681.7; [M+13H]13+=625.4. The experimental mass agrees with the theoretical mass of 7494.23 Da [M+1].

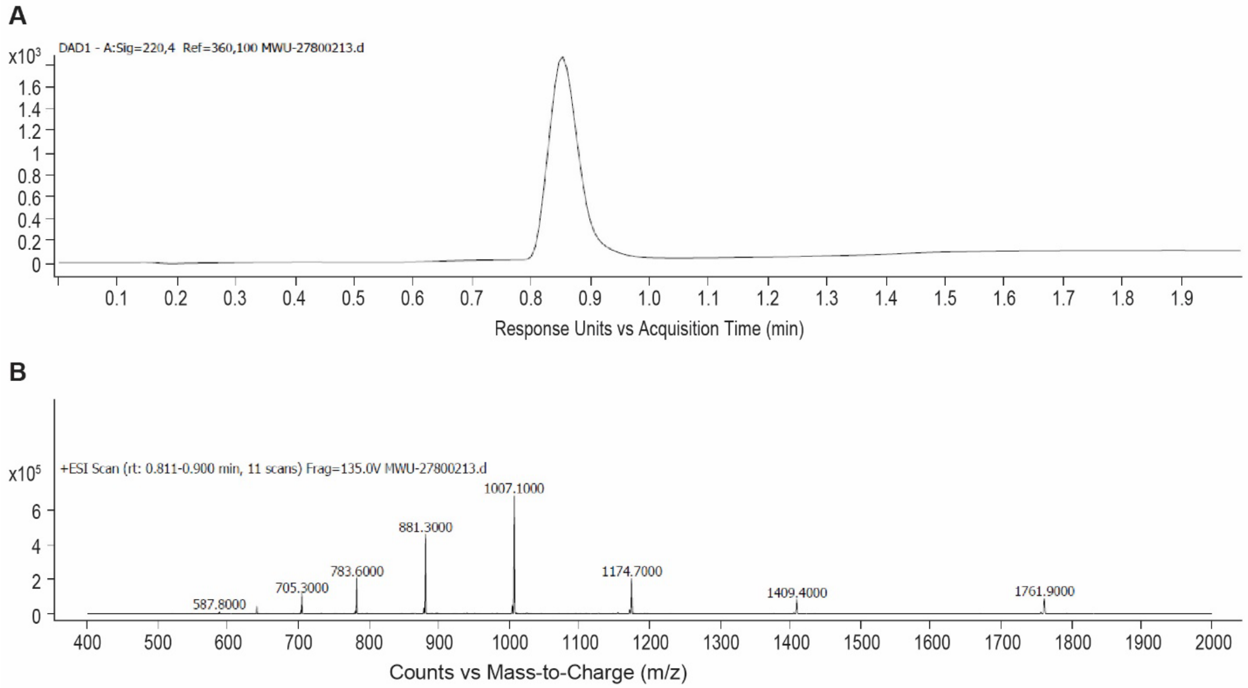

Figure S3. Characterization of Clone 7 affibody: (A) UPLC chromatogram. (B) Mass spectrum of the purified peptide. Observed m/z values include: [M+4H]4+=1761.9; [M+5H]5+=1409.4; [M+6H]6+=1174.7; [M+7H]7+=1007.1; [M+8H]8+=881.3; [M+9H]9+=783.6; [M+10H]10+=705.3; [M+11H]11+=641.3; [M+12H]12+=587.8. The experimental mass agrees with the theoretical mass of 7041.84 Da [M+1].

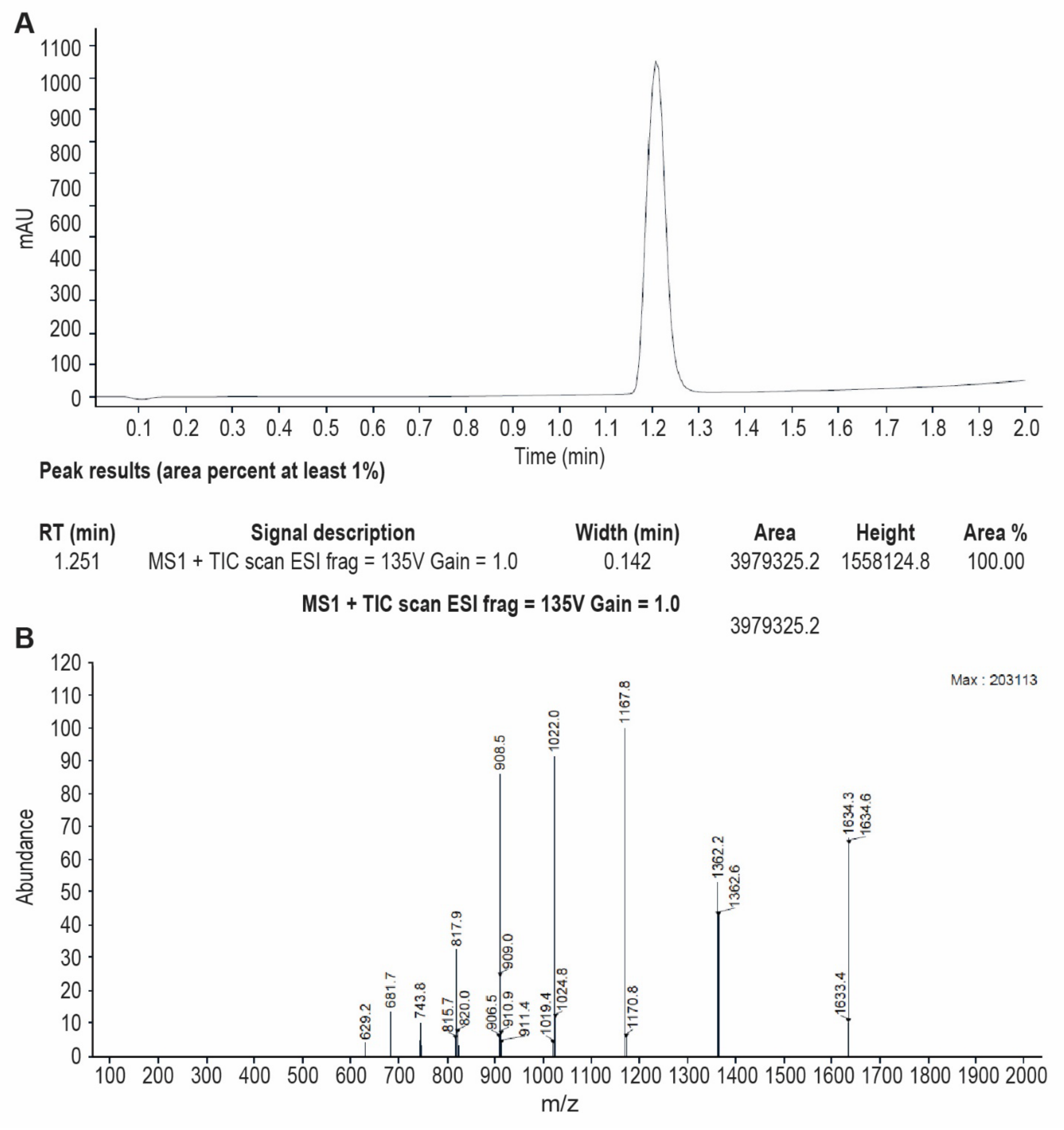

Figure S4. Characterization of Clone 7-Dy800 affibody: (A) UPLC chromatogram. (B) Mass spectrum of the purified peptide. Observed m/z values include: [M+5H]5+=1634.6; [M+6H]6+=1362.2; [M+7H]7+=1167.8; [M+8H]8+=1022.0; [M+9H]9+=908.5; [M+10H]10+=817.9; [M+11H]11+=743.8; [M+12H]12+=681.7; [M+13H]13+=629.2. The experimental mass agrees with the theoretical mass of 8167.05 Da [M+1].

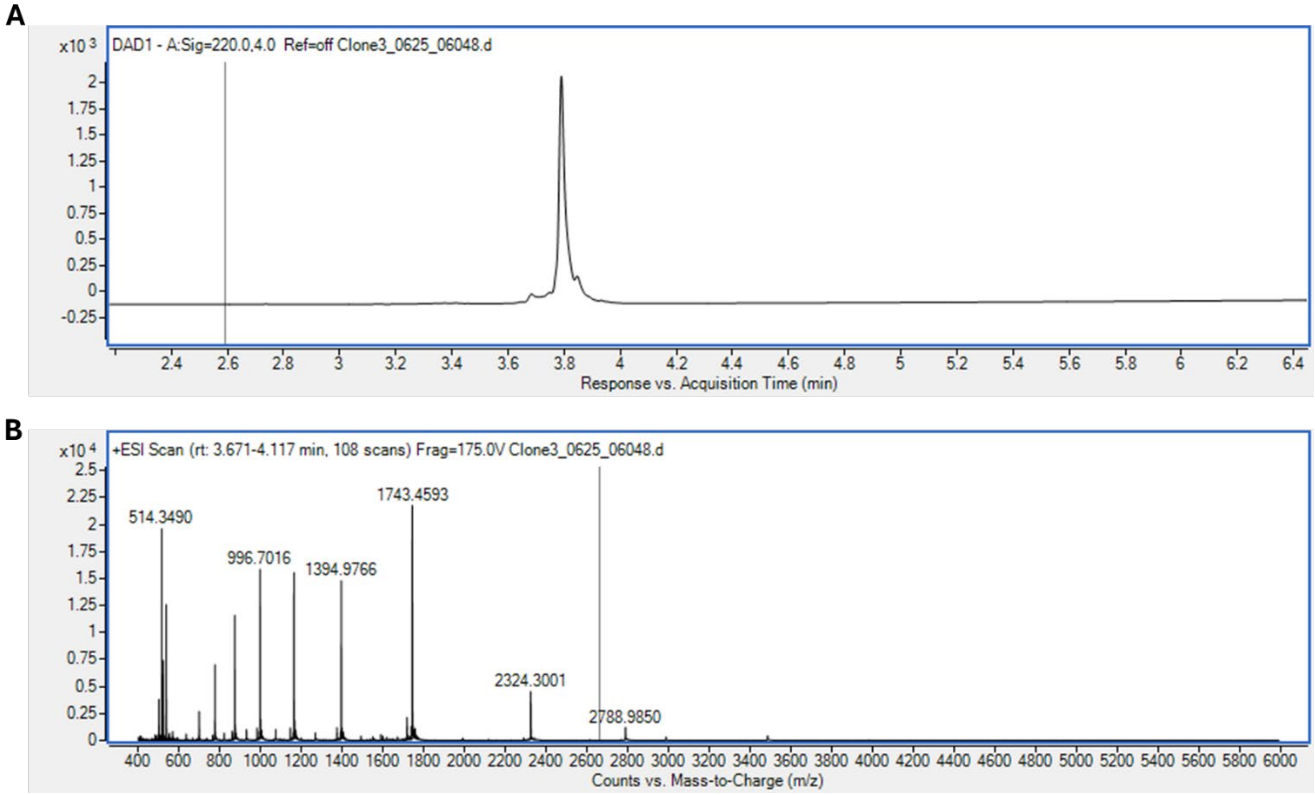

Figure S5. Characterization of Clone 3 affibody: (A) UPLC chromatogram. (B) Mass spectrum of the purified peptide. Observed m/z values include: [M+3]/3=2324.30; [M+4]/4=1743.45; The experimental mass agrees with the theoretical mass of 6927.64 Da.

A

B

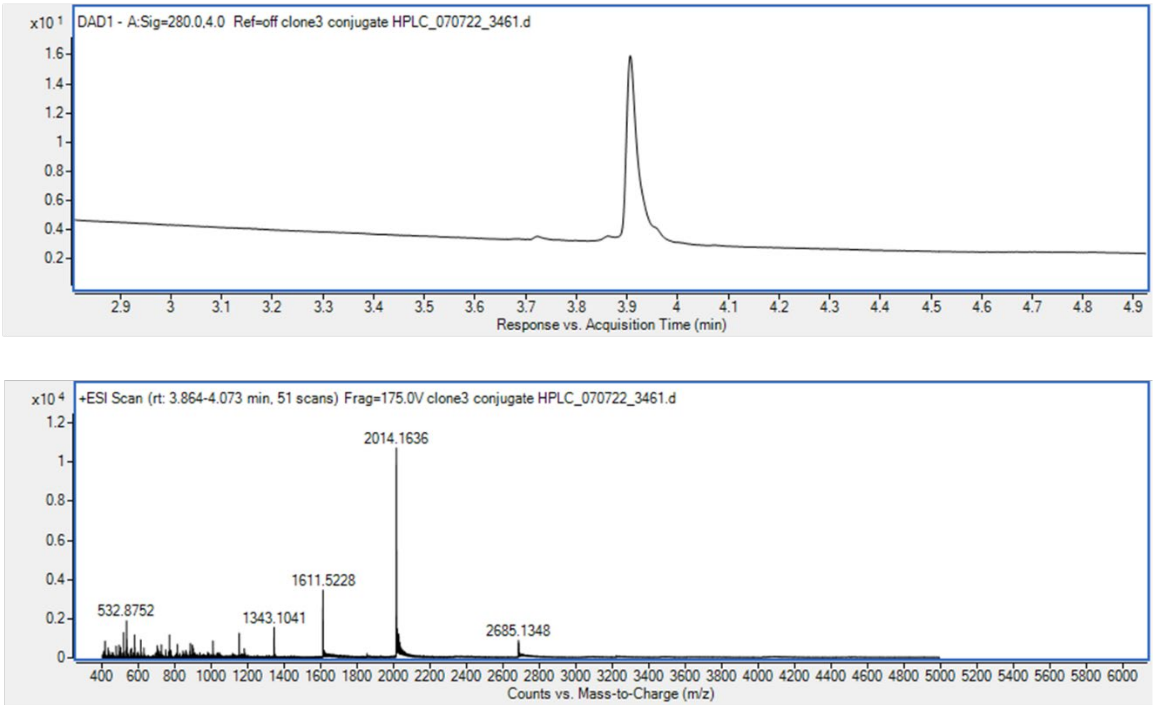

Figure S6. Characterization of Clone 3-Dy800 affibody: (A) UPLC chromatogram. (B) Mass spectrum of the purified peptide. Observed m/z values include: [M+3]/3=2685.13; [M+4]/4=2014.16. The experimental mass agrees with the theoretical mass of 8053.96 Da.

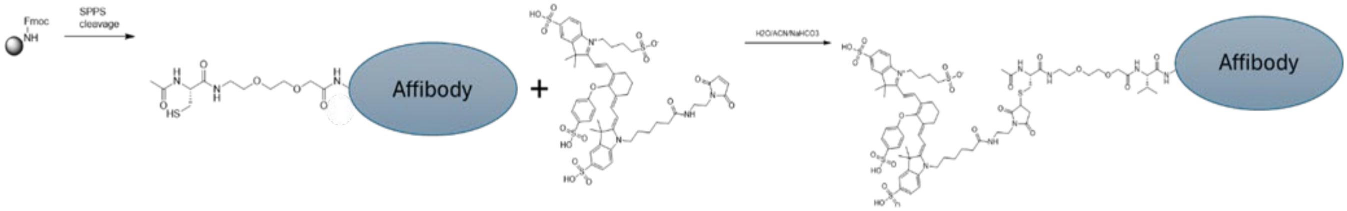

Schematic S1. Representation of the affibody conjugated to Dy800. The affibody backbone is shown in dark teal. A short PEG spacer was introduced at the N-terminus to enhance solubility, and a cysteine residue was incorporated into the PEG linker to allow site-specific conjugation with the maleimide-functionalized Dy800 dye.

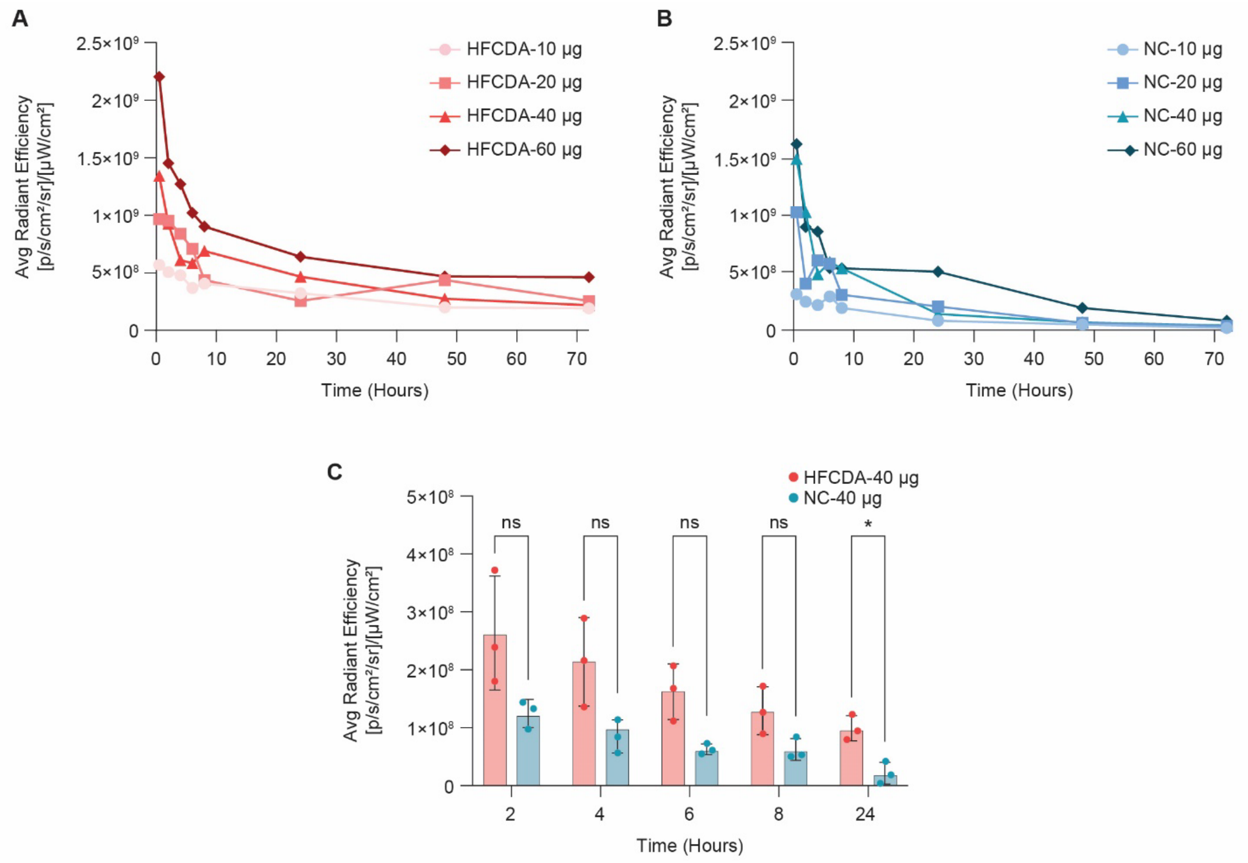

Figure S7. (A) In vivo biodistribution at different dosages of clone 3-Dy800 in HFCDA. (B) NC fed mice (n=1 mouse per dosage). (C) In vivo biodistribution of Clone 3-Dy800 at 40µg up to 24 h (n=3 for NC and n=3 for HFCDA mice) (*P<0.038 and nsP<0.10).

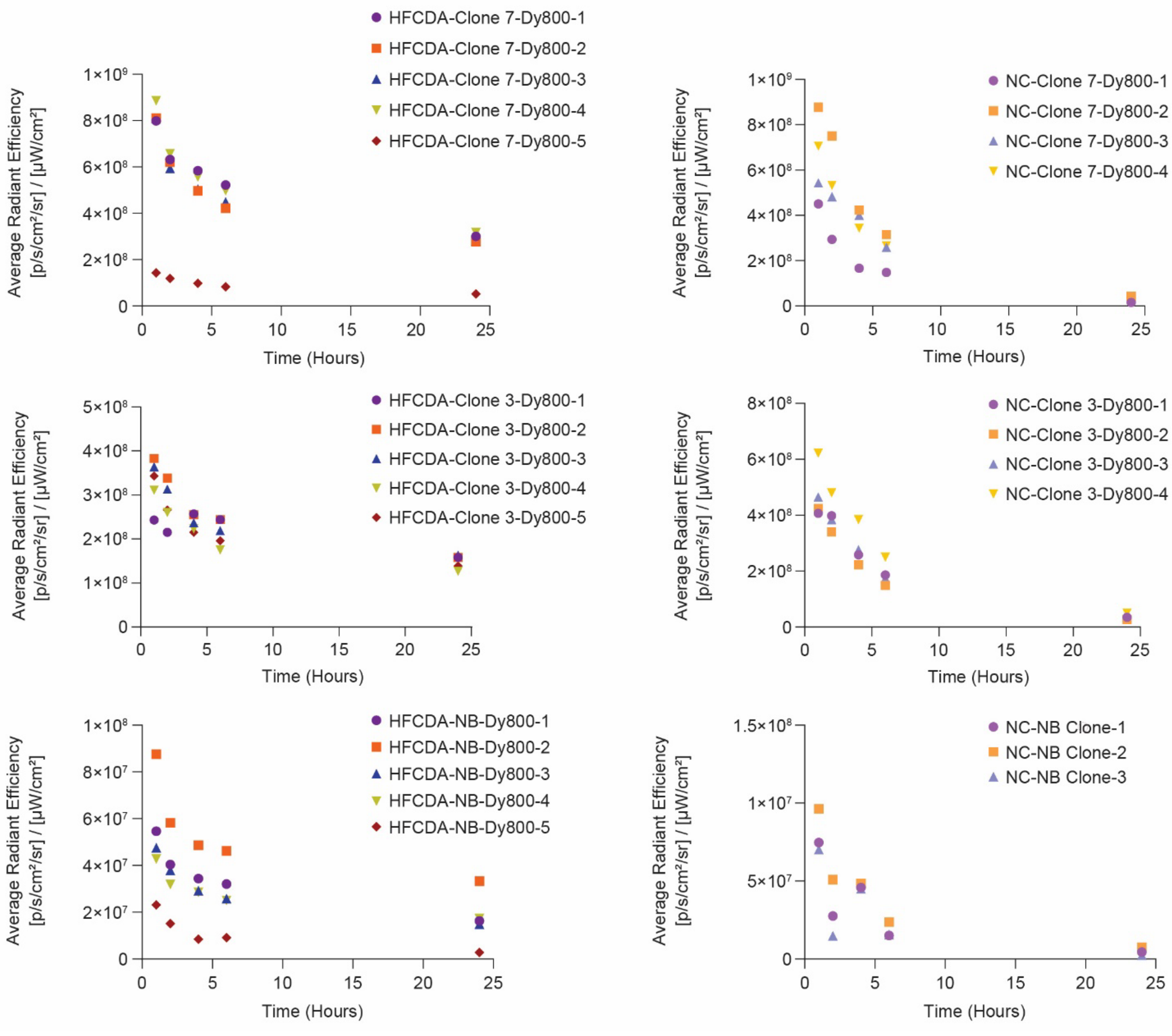

Figure S8. In vivo biodistribution results of Clone 7-Dy800, Clone 3-Dy800, and NB-Dy800 probes in HFCDA and NC-fed mice at different time points (1, 2, 4, 6, and 24 hs). Data were collected by drawing a ROI of the same size from the chest area and reported as average radiant efficiency for each individual mouse.

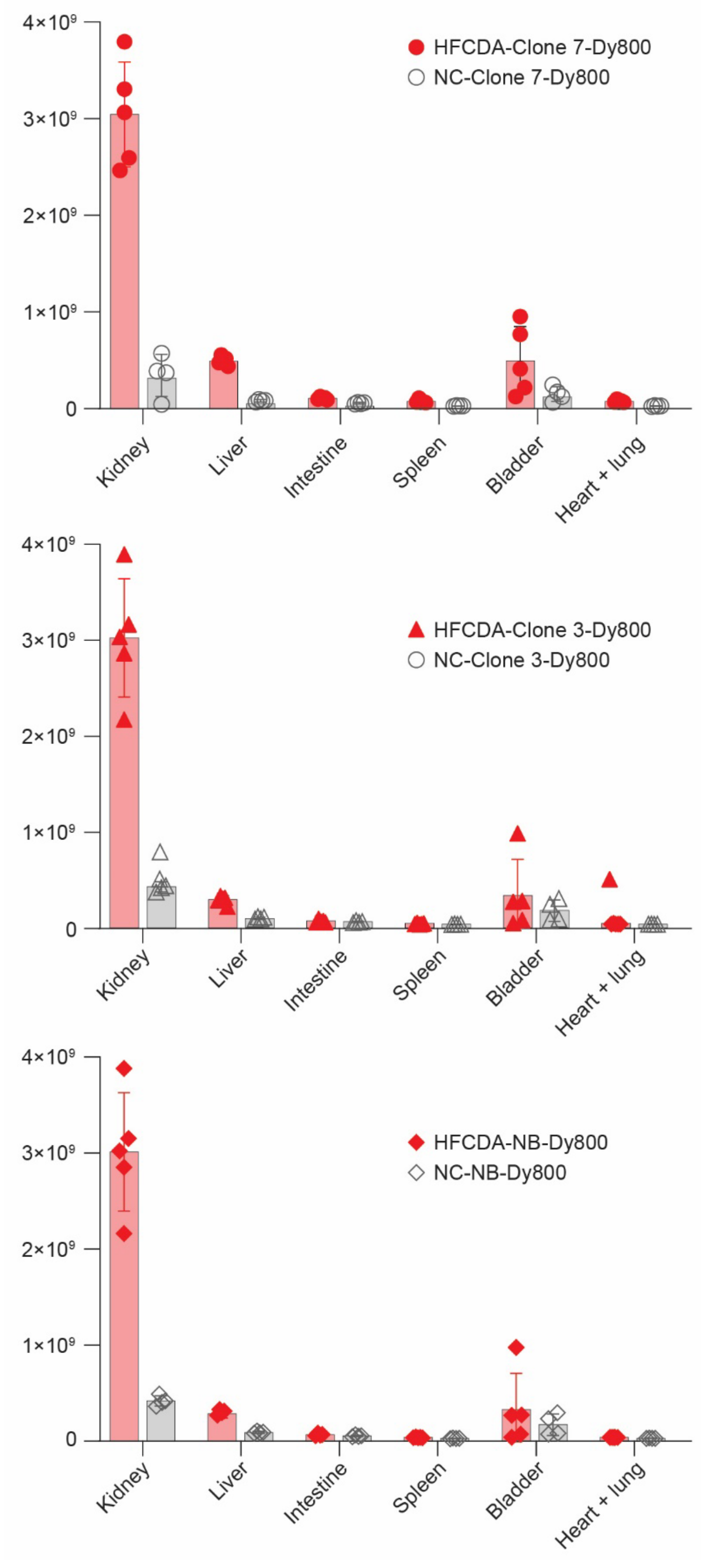

Figure S9. Ex vivo quantitative analysis of the fluorescence signal of the major organs of HFCDA and NC mice at 24 hr post clone 7-Dy800 injection (HFCDA=5, NC=4), clone 3-Dy800(HFCDA=5, NC=4), and NB-Dy800(HFCDA=5, NC=3).

Table S1: Quantitative histological assessment of liver sections from GAN and NC animal groups.

Animal ID	% DAPI Area	% COL-1 Area	NAS	PSR%
GAN-1	10.19%	1.51%	7	22.10647
GAN-2	18.02%	4.40%	5	33.07903
GAN-3	11.70%	3.12%	7	28.42713
NC-1	11.80%	0.62%	6	3.853449
NC-2	10.67%	0.86%	3	6.960868

Table S2. Correlation of Clone7-Dy800 fluorescence signal with histological fibrosis markers in HFCDA and NC animal groups.

Animal ID	COL-1 (%Area)	PSR%	NAS	Avg Radiant Efficiency
HFCDA-1	20.4339	10.2061	5	5.69e+008
HFCDA-2	18.0652	7.4323	4	4.75e+008
HFCDA-3	19.5896	7.5646	6	4.67e+008
HFCDA-4	19.0784	7.9078	6	5.09e+008
HFCDA-5	17.0208	6.7515	4	4e+008
NC-1	3.4634	2.0904	0	1.17E+08
NC-2	4.7203	2.7591	2	1.20E+08
NC-3	3.6755	2.5446	1	1.37E+08
NC-4	5.0798	2.1580	2	1.12E+08
NC-5	3.5718	2.6922	2	1.38E+08

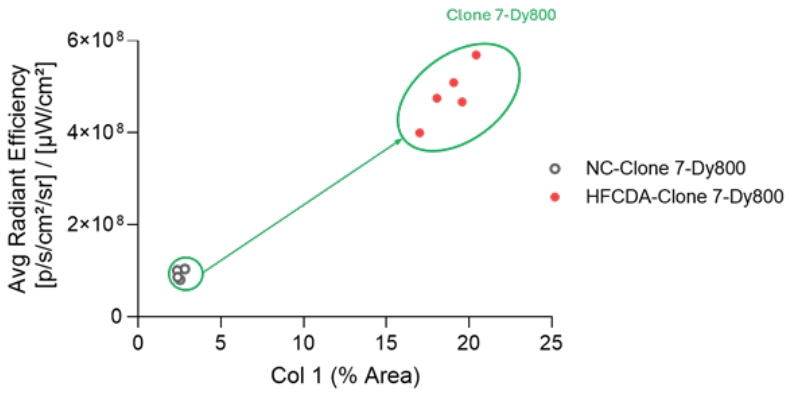

Figure S10. Correlation between liver signal intensity from the clone 7-Dy800 and COL-1 expression level in HFCDA and NC animal models (r^2=0.77).

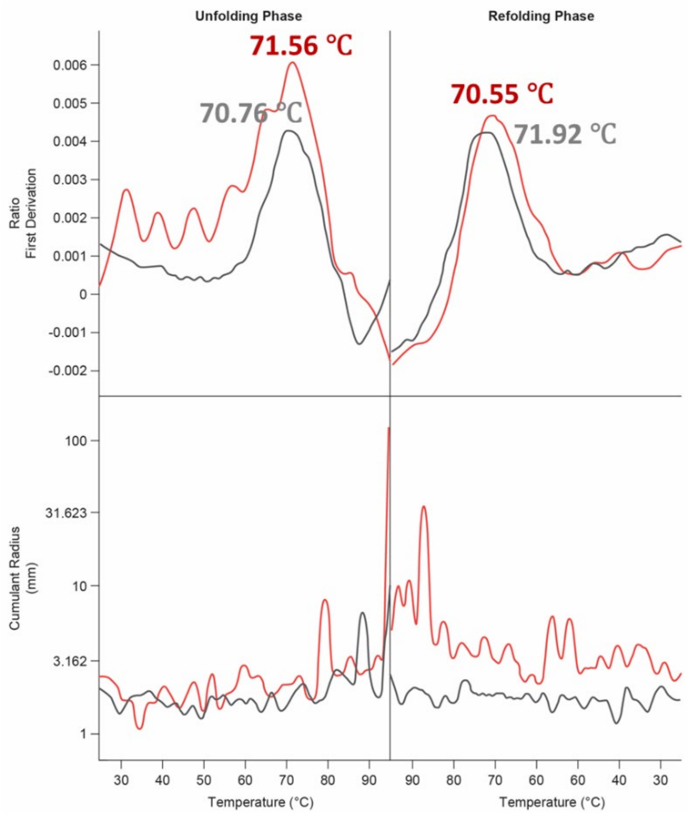

Figure S11. Thermal stability and size distribution analysis of clone7 and clone7-Dy800. Top panels: Thermal unfolding and refolding profiles of clone7 (gray color) and clone7-Dy800 (red color) measured using the Prometheus Panta system. Both clones exhibit high thermal stability with a melting temperature (Tm) of ~71°C. Bottom panels: Dynamic light scattering (DLS) measurements showing monodisperse size distributions for bothcClone7 and clone7-Dy800, with no signs of aggregation.
